# On Simulating Neural Damage in Connectionist Networks

**DOI:** 10.1007/s42113-020-00081-z

**Published:** 2020-06-30

**Authors:** Olivia Guest, Andrea Caso, Richard P. Cooper

**Affiliations:** 1 Research Centre on Interactive Media, Smart Systems and Emerging Technologies — RISE, Nicosia, Cyprus; 2 Department of Experimental Psychology, University College London, London, UK; 3 Center for Cognition, Computation and Modelling, Department of Psychological Sciences, Birkbeck, London, UK

**Keywords:** Connectionism, Connectionist cognitive neuropsychology, Semantic cognition, Sequential action selection, Methodology, Replication

## Abstract

A key strength of connectionist modelling is its ability to simulate both intact cognition and the behavioural effects of neural damage. We survey the literature, showing that models have been damaged in a variety of ways, e.g. by removing connections, by adding noise to connection weights, by scaling weights, by removing units and by adding noise to unit activations. While these different implementations of damage have often been assumed to be behaviourally equivalent, some theorists have made aetiological claims that rest on nonequivalence. They suggest that related deficits with different aetiologies might be accounted for by different forms of damage within a single model. We present two case studies that explore the effects of different forms of damage in two influential connectionist models, each of which has been applied to explain neuropsychological deficits. Our results indicate that the effect of simulated damage can indeed be sensitive to the way in which damage is implemented, particularly when the environment comprises subsets of items that differ in their statistical properties, but such effects are sensitive to relatively subtle aspects of the model’s training environment. We argue that, as a consequence, substantial methodological care is required if aetiological claims about simulated neural damage are to be justified, and conclude more generally that implementation assumptions, including those concerning simulated damage, must be fully explored when evaluating models of neurological deficits, both to avoid over-extending the explanatory power of specific implementations and to ensure that reported results are replicable.

**Electronic Supplementary Material:**

The online version of this article (10.1007/s42113-020-00081-z) contains supplementary material, which is available to authorized users.

## Introduction

Following a surge of interest in the 1980s and 1990s, connectionism, in which behaviours of interest are simulated by networks of computationally simple units which pass activation to each other in parallel via weighted connections, has become a standard approach within cognitive modelling. An important strength of the approach is that it can provide insights into how neuropsychological deficits (i.e. behavioural impairments following neural damage) might arise from damage at a computational or information processing level. For example, in one of the earliest applications of the technique Hinton and Shallice (1991, first published in 1989 as a technical report) showed how, when damaged, a connectionist model of reading exhibited characteristics of acquired dyslexia, similar to certain groups of neurological patients. Damage was implemented in the model in three distinct ways: through severing a small proportion of weighted connections; by removing a small proportion of units; and by perturbing activations through the addition of random noise.

A successful connectionist neuropsychological simulation can both provide support for the cognitive theory implemented within the simulation and strengthen our understanding of functional deficits underlying relevant behavioural impairments. In this way, connectionism appears to offer a level of abstraction that allows it to capture both cognitive theory and the effects of neural damage within that theory. Consequently, connectionist models allow us to combine theories regarding computational mechanisms with both high-level neuropsychological and behavioural investigations and lower-level lesion, neuroimaging, and neurophysiological studies. Thus, a key strength of the connectionist approach is that it bridges levels.

The general method of simulating neuropsychological deficits within a connectionist model involves first training the model on a set of patterns held to reflect the input/output regularities of a domain, “damaging” the model to simulate the neurological impairment of interest, exploring the effects of that damage on the model’s behaviour, and finally extracting implications of the simulation work for cognitive-level theory. This general method has been used to simulate a wide range of deficits including various forms of agnosia, aphasia, semantic impairments, dysgraphia and dyslexia, and the simulation work has supported theoretical developments in our understanding across numerous areas of human cognition, including language, memory, sequential action selection and object knowledge.

At the same time, the general method has been applied across the various areas in several different ways. As noted above, Hinton and Shallice (1991) considered three different implementations of damage, but many authors consider just one implementation of damage. For example, Plaut and Shallice (1993b) report a model of naming errors in optic aphasia where damage to the model is implemented through severing of connections between units, while Tippett and Farah (1994) report a model of naming deficits in Alzheimer’s disease where damage is implemented through removal of units. Others have implemented damage through adding noise to connection weights, adding noise to the activation of units or reducing the relative strengths of weights by a fixed factor. (See below for further details.) This paper is concerned with the relations between these different methods of implementing damage within connectionist models, and with whether different methods of implementing damage (within a single model) might be reasonably related to different neural pathologies.

### Common Implementations of Damage

To illustrate the range both of neuropsychological impairments and of implementations of damage, Table 1 summarises the specific methods adopted by some of the more influential research in a selection of areas. Rows in the table refer to broad kinds of impairment, e.g. agnosia in the first row, while columns correspond to the method of implementation of damage used to affect the healthy functioning of the trained models, e.g. connection severing. The rows of the table are deliberately broad, e.g. agnosia has several subtypes, including auditory, visual and tactile, and can be specific to certain kinds of stimuli, as in prosopagnosia. Moreover, models within each row can capture widely different types of neuropsychological deficit. Nevertheless, Table 1, which highlights five distinct approaches to implementing damage, demonstrates that there has been considerable variability in the way in which damage has been implemented.

**Table 1:**
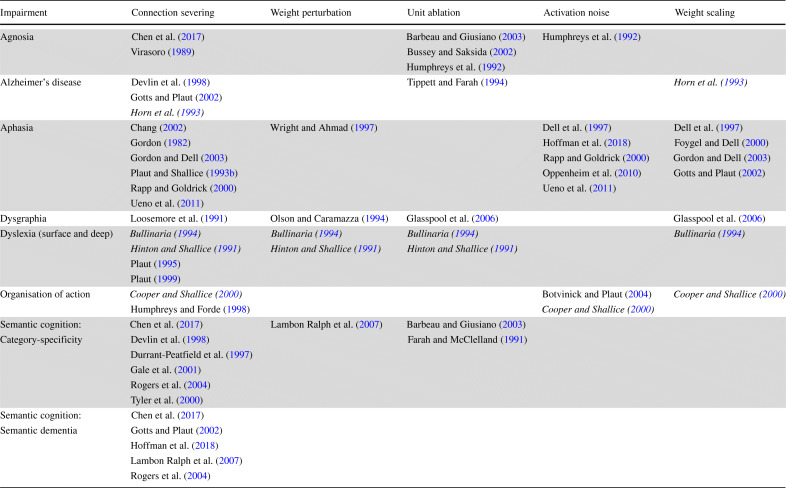
Survey of various connectionist models of deficits

The table above presents a sample of the literature of connectionist models of neuropsychological deficits both in terms of the type of damage (columns) used in the model and the kinds of impairments (rows) the models aim to capture. While only presenting a subset of the work published in this area, the table gives a bird’s eye view of the type of work carried out in terms of, for example, different types of damage used to model the same groups of patients or behaviours (shown in italics in the table: Bullinaria 1994; Cooper and Shallice 2000; Hinton and Shallice 1991; Horn et al. 1993). Note also that some models (e.g. Dell et al. 1997 and Cooper and Shallice 2000) have considered, in addition to the above, increased decay of activation as a proxy for neural damage

The first approach considered in the table, connection severing, involves setting a proportion, *q*, of weights to zero (or more formally, multiplying each weight by a random variable drawn from a Bernoulli distribution with parameter 1 − *q*, where *q* is the probability of a weight being severed). Connection severing can be applied across all connections in the network (e.g. Rogers et al. 2004) or it can be targetted at, or to discover, specific pathways within a model (e.g. Yang et al. 2019), which may be identified with neural pathways.

The second form of damage, perturbing network weights, is most commonly implemented by adding Gaussian noise with predefined variance *σ*^2^ to all weights between layers. A third possibility is unit ablation, where the weights of all connections from a proportion, *q*, of units are set to zero, meaning that a proportion of units makes no contribution to downstream processing. Ablating units is equivalent to severing all outgoing connections from those units. Paralleling the case of removing connections, unit ablation is typically performed by, for each unit, drawing a random variable from a Bernoulli distribution with parameter 1 − *q*, where *q* is the probability of a unit being ablated, and multiplying all output weights of the unit by this variable.

The fourth possibility considered in the table is the addition of Gaussian noise (with predefined variance *σ*^2^) to the activations of units. Within layered networks, this can be thought of as blurring the N-dimensional representations on a layer. This contrasts with ablating units, which can be seen as losing components or micro-features of the representations altogether.

Finally, all weights within a network may be scaled by a factor *s*, where *s* is less than one but greater than zero. Weight scaling can be seen as applying a generalised form of dampening to a network’s activity, but it is functionally equivalent to altering the gain of the activation function, i.e. the function that maps the summed weighted input of a unit to its activation. The latter has been used, for example, to model dysfunction of neuromodulation and its hypothesised effects (e.g. Gotts and Plaut 2002).

Three general points that apply across the various forms of damage can be made. First, for each type of damage, there is a single parameter that determines the level of damage, and this parameter is normally varied to capture differing severities of impairment. Second, with the exception of weight scaling, lesioning a network at any specified level is non-deterministic, in the sense that a given network may be lesioned at a given level in multiple distinct ways. Consequently, when exploring the effects of damage on a network, it is necessary to lesion the network multiple times at each level and determine mean performance at each level. Third, in networks where connection weights are learned, all units typically include input from a bias unit, which is always active. The bias weight to each unit determines that unit’s firing threshold, and is thus critical for establishing the network’s behaviour in the absence of input. Bias units and their weights are normally assumed to be unaffected by damage. In line with this practice, in all cases considered here, bias units and their weights are exempt from damage.

### Behavioural Consequences of the Implementation of Damage

While not all forms of damage are appropriate for all networks, early work suggested that different ways of imposing damage on a network (or on a pathway within a network) had essentially the same behavioural consequences. Thus, in their seminal work on modelling deep dyslexia, Plaut and Shallice (1993a) considered three different types of damage (namely severing of connections, ablation of units and adding noise to weights). All three forms of damage were shown to result in equivalent functional deficits. The authors concluded that the critical feature of their model was the presence of attractor states—stable states that the model tended towards with constant input—within their model’s orthographic, semantic and phonological domains. Damage within the model, whatever form it might take, was argued to distort the model’s attractors such that a given input might ultimately lead to an inappropriate, but related, attractor state (see also Hinton and Shallice 1991).

Similar results with respect to the invariance of network behaviour with respect to the type or implementation of damage were presented by Bullinaria and Chater (1995), who looked at the ability of feed-forward networks to capture rules with exceptions. The authors compared five types of damage: scaling of weights (i.e. multiplying all weights by a constant between 0 and 1), reducing weights (i.e. subtracting a constant from all weights), adding Gaussian noise to all weights, removing hidden units, and removing connections. Bullinaria and Chater (1995) concluded that differences between the types of damage reflect the random factors involved in applying them, and that in larger networks scaling of weights is the least noisy approximation of the other types of damage. Along the same line of inquiry, Humphreys et al. (1992) showed in a model of visual search that the same functional deficit can arise as a result of at least two different types of lesion: perturbing weights and ablating units.

In line with the findings mentioned above (i.e. Humphreys et al. 1992; Bullinaria and Chater 1995; Hinton and Shallice^1991^; Plaut and Shallice 1993a), connectionist neuropsychological accounts have tended to assume without comment that the way in which damage is implemented does not affect network behaviour in any critical way. Moreover, while there are some exceptions (e.g. Bullinaria 1994; Cooper and Shallice 2000), most reports model damage by disconnecting units or by setting their connection weights to zero, and do not present the various forms of damage as meaningful contrasts within a model.

However, these early results appear to be at odds with more recent work by Lambon Ralph et al. (2007), who argued that two different progressive neural pathologies that may affect the semantic system, but which result in different behavioural deficits, might be modelled by different forms of simulated damage within a single connectionist network. While the most recent work from this group advances an alternative account of the different pathologies of semantic cognition (Chen et al. 2017), the claim concerning the relation between implementation of damage and aetiology continues to have currency within the broader cognitive neuropsychological literature (e.g. Seckin et al. 2016). The claim is also implicit in models involving other neuropsychological domains. Thus, Ueno et al. (2011) lesion their “Lichtheim 2” model of the neural basis of language by simultaneously severing connections and adding noise to weights, on the explicit assumptions that (a) the former is analogous to white matter damage while the latter is analogous to grey matter damage and (b) most patients of relevance to the model have both white and grey matter damage. The veracity of the first of these assumptions is not questioned. Just as critically, Ueno et al. (2011) do not consider whether their simulation results are dependent on these assumptions.

### The Structure of this Article

Given this context, in this paper, we seek to evaluate the extent to which different forms of generalised damage within connectionist networks are, or are not, behaviourally equivalent. The issue is not just one of understanding the basic principles of connectionist accounts. It is critical in understanding whether the implementation of damage within a model is of theoretical interest and hence the extent to which connectionist accounts of neuropathology can be linked to aetiology. For example, if a model’s behaviour following damage depends on the way in which damage is implemented, then the implementation of damage becomes an essential element of the theoretical account of the neuropsychological deficit under consideration. If, on the other hand, a model’s behaviour following damage does not depend in any substantive way on the method of implementing damage, then that aspect of the account may legitimately be abstracted away when theorising about the relevant cognitive processes and impairments. In other words, if behaviour following damage depends on the form of damage, then we require bridging assumptions linking the connectionist model and the neural level. If it does not, then such assumptions are unnecessary, and arguably irrelevant, and theorising can proceed entirely at an information-processing level.

To consider these issues, we first examine the arguments and model of Lambon Ralph et al. (2007). We present two replications of that work, comparing the effects of different implementations of damage on naming different types of stimuli within their model of semantic cognition. Moreover, we extend their study by considering the effects of four separate forms of damage (including two forms not previously considered: ablation, or unit removal, and weight scaling). One of our two replications was successful, with connection severing producing a generalised naming impairment but weight perturbation having a greater effect on animal naming than artefact naming, as in the original model. However, the other replication was not. We therefore consider why the results of our replications were inconsistent. To foreshadow those considerations, we demonstrate that the findings of Lambon Ralph et al. (2007) are a consequence of statistical properties of the specific set of items on which their network was trained—properties that do not hold of the items used in their earlier work with the same model (i.e. that of Rogers et al. 2004). This has significant implications for that original simulation work, as it suggests that the earlier and subsequent results may depend upon different assumptions concerning the training set. More critically, we show that when the network is trained with the original training set of Rogers et al. (2004) and then damaged through the severing of connections, it produces the reverse dissociation (greater difficulty with artefact naming than animal naming). Thus, equating connection severing and weight perturbation with specific distinct aetiologies, as suggested by Lambon Ralph et al. (2007), is problematic.

We then explore the effects of different types of damage with a second case study based on a second model within the broad family of distributed learning network models, namely the simple recurrent network (SRN) model of routine sequential behaviour and its disorders presented by Botvinick and Plaut (2004). In this case, five forms of damage are considered (the previous four, plus activation noise—i.e. all five forms of damage mentioned in Table 1). Echoing the position in previous work (Humphreys et al. 1992, Bullinaria and Chater 1995, Hinton and Shallice 1991, and Plaut and Shallice 1993a), we demonstrate that in this model all forms of damage except weight scaling are functionally equivalent.

The difference between the cases, where different forms of damage do or do not result in different behavioural consequences, depends upon the degree to which critical behaviours are driven by statistical regularities in the model’s input. In the model of Lambon Ralph et al. (2007), the different effects of different forms of damage depend upon two specific statistical properties of the model’s training patterns (one concerning the relative co-occurrence of features in different subsets of the training patterns, as originally argued by the authors, and another concerning differences in the mean number of features within each of the subsets of the training patterns, a difference not discussed in the original work). In the second case study, such properties play no direct role in the model’s behaviour following damage.

These conclusions, which are further bolstered by additional simulations reported in Supplementary Materials, have several important consequences. For example, suppose that, in some specific cognitive domain, one can establish differential effects of different types of damage (as argued for semantic cognition by Lambon Ralph et al. 2007). Such a pattern would seem to imply that the explanation of such effects should be sought more in regularities in the input to the relevant cognitive systems (or model thereof) than in the system itself. Conversely, the lack of such an interaction (or equivalently the independence of behaviour and type of damage) has implications for the nature of the training patterns (i.e. those patterns should not show statistical differences that might interact with type of damage). More generally, without full investigation, the implementation of damage to a connectionist model cannot be assumed to be an irrelevant implementation detail. Equally, nor can it be appealed to in support of a cognitive theory without consideration of further aspects of the combined model plus training set which might modulate any apparent effects of type of damage.

## Case Study 1: Lesioning the Hub-and-Spoke Model of the Organisation of Semantic Knowledge

### Category-Specific Impairments of Semantic Knowledge

In a now classic study, Warrington and Shallice (1984) described four herpes simplex virus encephalitis (HSVE) patients who, when tested with a word-picture matching task, had great difficulty in identifying or naming living things, e.g. ⌜*spider*⌝^1^ or ⌜*duck*⌝, while generally performing less poorly when identifying or naming inanimate objects, e.g. ⌜*umbrella*⌝ or ⌜*wheelbarrow*⌝. Subsequent work (e.g. Lambon Ralph et al. 2007; Noppeney et al. 2007) has found that, broadly speaking, while HSVE patients have a generalised semantic impairment, that impairment is typically more severe for some types of object (notably living things) than others (notably artefacts).

While many studies have reported similar dissociations between knowledge of animals and knowledge of inanimate objects, the reverse dissociation—with better performance on inanimate than animate objects—has also been observed. For example, Warrington and McCarthy (1983, 1987) report two stroke patients (one with left frontoparietal damage and another with left temporoparietal damage) who performed better with living things than inanimate objects on a word-picture matching task (see Capitani et al. 2003, for a comprehensive review of the relevant cases reported up to 2003, and Campanella et al. 2010, for a more recent group study of left posterior temporal tumour patients showing a similar dissociation). Deficits such as these provide insight into the structuring of, and mechanisms of access to, human semantic knowledge, and numerous accounts of what has come to be known as “semantic cognition” have been proposed in order to account for one or more of these deficits (see Caramazza and Mahon 2003, for a review).

The deficit resulting from HSVE is of particular interest due to its contrast with that of semantic dementia (SD; Snowden et al. 1989) patients. Like HSVE patients, SD patients typically have bilateral lesions affecting the anterior temporal lobe, and while the affected regions are not identical, SD patients, like HSVE patients, show impairments of semantic knowledge. Thus, SD patients perform poorly on many classic tests such as picture naming, word sorting and picture sorting. However, SD patients generally do not show the sensitivity to category often shown by HSVE patients. The SD deficit is typically more general (Hodges et al. 1992; though see also Libon et al. 2013). Accounting for these patterns of impairment—that of SD, HSVE and impairments primarily affecting artefact knowledge—within a single framework is thus of major theoretical importance.

### The Hub-and-Spoke Model of Semantic Cognition

**Fig. 1:**
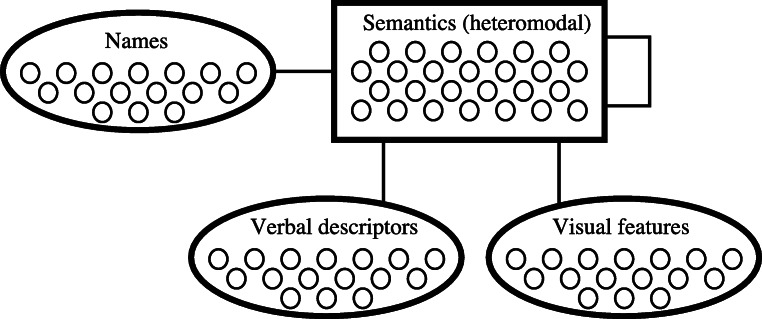
A high-level depiction of the architecture of the hub-and-spoke model showing the hub (semantic units) bidirectionally connected to the modality-specific spokes: name, verbal and visual units (adapted from Fig. 2, Lambon Ralph et al. 2007)

One account of the differences between the semantic abilities of SD and HSVE patients is that of Lambon Ralph et al. (2007). The account is based on the “hub-and-spoke” model of semantic cognition, first proposed by Rogers et al. (2004; see also Lambon Ralph et al. 2017), within which semantic knowledge is represented in an amodal “hub” that is accessed via modality-specific “spokes” (see Fig. 1). Modality-specific knowledge relating to visual, verbal, auditory etc. features of a concept is combined, or abstracted over, within an amodal representation that arises in the central hub. These representations (both modal and amodal) may be accessed (and reactivated) via input from any modality or combination thereof. Critically, a similarity function operates over amodal representations, such that entities that are similar across modalities have similar amodal representations. Therefore in the representational space of the hub, as is the case in the majority of modality-based input spaces, ⌜*tomato*⌝ is closer to ⌜*apple*⌝ than to ⌜*robin*⌝, but closer to ⌜*robin*⌝ than to ⌜*hammer*⌝.

Lambon Ralph et al. (2007) put forward the novel proposal that the behavioural profiles of SD and HSVE patients on tests of semantic cognition are due to a difference in the type, but *not* in the locus, of neural damage (as has been proposed by others, e.g. Noppeney et al. 2007). More specifically, they argue that SD is associated with the “dimming” of knowledge, such that closely related concepts (which have few distinguishing features) merge into one more general concept. In contrast, they hold that HSVE reflects “distortion” of semantic knowledge, such that “similar representations are easily confused” (Lambon Ralph et al. 2007, p. 1135). Dimming is argued to have similar effects across a variety of categories (meaning it should not yield category-specific deficits). On the other hand, distortion, because of its nature, is held to particularly affect categories with few distinguishing features (or equivalently, predominantly common features), such as animals. As a consequence, distortion yields specific deficits related to knowledge of living things. The contrast between distortion and dimming within the hub-and-spoke model is the crux of the Lambon Ralph et al. (2007) account of the differing semantic deficits of SD and HSVE patients.

The hub-and-spoke model is implemented as a recurrent connectionist network, with an architecture as shown in Fig. 1. The model is trained on patterns that are held to embody real-world feature co-occurrence statistics (e.g. relating to 〈*has eyes*〉 and 〈*can see*〉) and that are derived from lists of properties for concepts generated by neurologically healthy participants (Garrard et al.^2001^). Training the network involves adjusting the bidirectional connection weights between the modality-specific interface units and the amodal hidden units. Over the course of training, semantic knowledge becomes encoded in a distributed fashion across the connection weights of the network. Subsequently, given an input in any one modality, activations propagate via the weighted connections throughout the network, giving rise to representations within the hub, labelled “Semantics (heteromodal)” in Fig. 1, and at the other interface units for each pattern.

Critically, Lambon Ralph et al. (2007) present simulations which show that the hub-and-spoke model produces a generalised SD-like semantic deficit when a random proportion of weights is set to zero. This is held to correspond to representational dimming. In contrast, the network produces a category-specific HSVE-like deficit, with relatively poorer knowledge of living things, when the connection weights are perturbed through the addition of random noise. This is held to correspond to representational distortion. These simulations lend support both to the hub-and-spoke theory of semantic cognition and to the associated account of the lower-level neuropathological origin of the difference between SD and HSVE deficits.

### Rationale for the Current Simulations

As noted in the introduction, more recent work derived from the hub-and-spoke approach has led to an alternative proposal of the behavioural difference between SD and HSVE, in which the differences in the deficits arise from damage to different pathways and selective relearning within an extended model (Chen et al. 2017), but the dimming/distortion account of the SD/HSVE contrast remains of interest from a theoretical perspective. Might, for example, dissociations in other cognitive domains be similarly accounted for by different forms of damage within a single model? Equally, might the reverse dissociation (of greater impairment on tasks involving knowledge of inanimate objects than animate objects), as observed in the patients reported by Warrington and McCarthy (1983, 1987), Capitani et al. (2003) and Campanella et al. (2010), be similarly explained by some other representational impairment corresponding to some other form of damage within an undifferentiated network?^2^

In fact, there are reasons to query the dimming/distortion account of the SD/HSVE contrast. For example, like most connectionist models, there is nothing to prevent the hub-and-spoke model (when trained) from having both positive and negative weighted connections. For a network with similar numbers of positive and negative weights, removal of connections is just as likely to result in increased activation in the network (the opposite of attractor dimming) as decreased activation. The addition of noise to weights is similarly likely to result in both increased activation of some units and decreased activation of other units. Thus, in order to be sure that removal or severing of connections in a network results in dimming (i.e. a reduction in the activation of stable states) rather than distortion, it is necessary to demonstrate that the distribution of weights is predominantly positive. However, pre-theoretically representational dimming would seem to be more plausibly modelled by scaling weights by some factor less than one. We return to these points in the “Results” and “Discussion” subsections of this section.

Therefore, in order to better understand the mechanisms underlying the hub-and-spoke model’s selective deficit for knowledge of living things following the introduction of weight noise, and as a starting point to explore how the theory might be extended to account for the reverse deficit, we set out to replicate the modelling results of Lambon Ralph et al. (2007). Two sets of simulations, using different sets of object/feature associations, were conducted: one with the pattern set distributed by McClelland (2015) with the open-source implementation of the hub-and-spoke model, and one with a series of pattern sets based on the specification given in the original description of the model (Rogers et al.^2004^).^3^

To foreshadow our results, only models trained on the former pattern set (i.e. those of McClelland, 2015) reproduced the results of Lambon Ralph et al. (2007). In fact, when the network was trained on the latter pattern set and weights were disrupted through the severing of connections, the model produced a selective deficit for artefact naming—more similar to the reverse deficit patients of Warrington and McCarthy (1983, 1987), Capitani et al. (2003) and Campanella et al. (2010) than to either HSVE or SD patients. Moreover, scaling of weights also led to behaviour that was highly dependent on the training set. In the remainder of this section, we describe our replication efforts and draw preliminary conclusions from our mixed success in reproducing the dissociation between loss of knowledge of animals and knowledge of objects as reported by Lambon Ralph et al. (2007).

### General Methods

The implementation of the hub-and-spoke theory, as originally described by Rogers et al. (2004) and employed in the simulations of Lambon Ralph et al. (2007), is a recurrent connectionist network with four sets of units (recall Fig. 1). One set represents verbal labels (e.g. “bird” or “cockatoo”), i.e. names. Another represents verbal descriptors, such as encyclopaedic knowledge, e.g. 〈*is found in Australia*〉, and taxonomic knowledge, e.g. 〈*is a bird*〉. A third set of units represents visual features, such as 〈*has two legs*〉. The final set, the semantic units, forms the amodal (or heteromodal) hub. The three other sets of units are bidirectionally fully connected to the hub units, while hub units are also connected to themselves (and each other) via recurrent connections.

The recurrent network is trained to auto-associate, for a set of objects and via the hub units, features presented at the name, verbal and visual sets of units. An established connectionist network training algorithm is used for this, namely “a variant of the backpropagation learning algorithm suited to learning in a recurrent network” (Rogers et al. 2004, p. 208).^4^

As noted above, we consider two distinct training sets: (a) the set distributed with the hub-and-spoke model in PDPTool, which we call P1, and (b) a set developed according to a template for generating compatible patterns described in Rogers et al. (2004), which we call P2.^5^ It is well known that the statistical properties of the training set are critical to learning within connectionist networks (e.g. McClelland et al. 2010). Therefore in both cases, we also explored the structure of the object descriptions to ensure that they adhered to contemporary understanding of object similarity, e.g. animals are more similar to each other than to non-living things, and so on (see Devereux et al. 2014; Garrard et al. 2001; McRae et al. 2005, for behavioural norms, and e.g. Kriegeskorte et al. 2008a, 2008b; Binder et al. 2016, for coarse evidence of the neural reality of such representational similarity for concrete objects). The models were then trained (as described below in the “Training and Lesioning” section) and “damaged”, either by setting a subset of connection weights to zero or by adding noise to all connection weights. For completeness, we also considered two further forms of damage discussed in the “Introduction” section but not considered by Lambon Ralph et al. (2007), namely complete removal of hidden (i.e. hub) units and scaling of weights. Finally, the performance of the differently trained and differently lesioned models was assessed on a simulated test of object naming, as in the study of Lambon Ralph et al. (2007).

It should be stressed that the original results of Lambon Ralph et al. (2007) were based on neither P1 nor P2, and it is not our aim to present a direct replication of this work or to question the simulation results presented in that paper. We will show below, however, that both P1 and P2 share the critical properties held by Lambon Ralph et al. (2007, see also Garrard et al. 2001; and Rogers et al. 2004) to distinguish animal and artefact domains, and held in that work to give rise to the different patterns of impairment (SD and HSVE) following different forms of neural and simulated damage.

### Characteristics of Pattern Sets

Both pattern sets comprise 48 items, with 216 binary features per item (40 name units, 64 visual units, 112 verbal units).

#### P1: PDPTool Patterns

Pattern set P1 was provided with the PDPTool model by McClelland (2015), one of the original authors of the hub-and-spoke model. The patterns within P1 represent items from six categories (birds, mammals, vehicles, household objects, tools and fruits), with 8 items from each category. The patterns are composed of binary vectors with features notionally representing properties such as 〈*is a mammal*〉, 〈*has fur*〉, 〈*can bark*〉 and 〈*is a pet*〉.

**Fig. 2:**
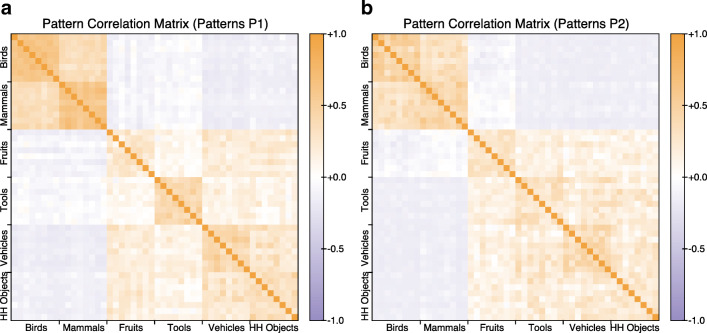
Correlation matrices showing the similarity space of the two training sets. The similarity space: **a** of pattern set P1, distributed with the PDPTool software; **b** of pattern set P2, generated from the template provided in Rogers et al. (2004). In both cases, there are strong within-category correlations (e.g. birds are more highly correlated with other birds than with anything else) and strong within-domain correlations (e.g. birds are more highly correlated with mammals than with artefacts of any type)

The similarity space of pattern set P1 is shown in Fig. 2a, in which each pattern is represented by a row and a column, with the cells in the grid representing the correlations between the corresponding patterns. The correlation matrices allow the structure that exists within the set of patterns, both at category-level (birds, mammals, vehicles, household objects, tools and fruit) and at domain-level (animate or inanimate), to be visualised. As can be seen from the figure, within each category, the patterns corresponding to the eight instances are positively correlated with each other, but within-category correlations are generally higher than between-category correlations, and within-domain correlations are higher than between-domain correlations. Importantly (from the theoretical perspective of the hub-and-spoke model), there is zero to slightly negative correlation between the two domains. That is, there are few, if any, features in common between exemplars from the two domains, thus ensuring that the input space is clearly partitioned into two subsets.

There is in fact a second difference between the patterns from each domain that is not captured in the correlation matrix. The mean number of features present (i.e. with value 1) per exemplar differs systematically across the categories and the domains, with more features present for animals than for artefacts (birds, 42.250; mammals, 41.375; fruits, 47.625; tools, 25.375; vehicles, 27.500; and household objects, 22.500). To some extent, this reflects findings from the empirical literature (e.g. Garrard et al. 2001; see also Tyler et al. 2000) where, in feature elicitation tasks, neurologically healthy individuals tend to list more features associated with living things (e.g. ⌜*elephant*⌝) than with non-living things (e.g. ⌜*hammer*⌝).

#### P2: Patterns Based on Rogers et al. (2004)

Pattern set P2 was created from the template provided in the original description of the hub-and-spoke model of Rogers et al. (2004). The template specifies the probability that each of 64 visual and 112 verbal features will be present for objects of each of the six different categories.^6^ For example, it specifies that the first visual feature will be absent for vehicles, tools, household objects and fruits, but that there is an 80% chance that the feature will be present for birds and mammals.

The correlation matrix of pattern set P2 is shown in Fig. 2b. The pattern set shares key properties with P1 concerning within-category and within-domain correlations. However the correlations within the birds and within the mammals are slightly weaker than in P1, as are the correlations between the birds and mammals. This means that birds and mammals are less densely packed categories than in P1.

Notwithstanding this seemingly minor difference, the two pattern sets have very similar representational structure. Thus, as with pattern set P1, more features are present for animals than for artefacts (birds, 35.875; mammals, 36.750; fruits, 31.500; tools, 24.250; vehicles, 28.625; and household objects, 24.625).^7^

Importantly, P2 is not atypical—it is a representative pattern set generated from the statistical template given by Rogers et al. (2004). As noted in footnote 5, to ensure that P2 is representative, we replicated our results with twenty different versions of P2 generated in the same way. The results presented below for P2 therefore follow from the statistical template rather than one specific but aberrant instance of patterns generated from it.

### Training and Lesioning

Twenty separate instances of the hub-and-spoke network from the PDPTool release (McClelland 2015), varying only in the initial random strength of connection weights, were trained on each pattern set. All networks were trained for 1000 epochs, with all patterns from a pattern set presented three times to the network on each epoch—once with the name units clamped, once with the verbal units clamped, and once with the visual units clamped—and weights updated after each pattern presentation. The PDPTool default values of weight decay (0.001) and learning rate (0.001) were used, following the instructions regarding convergence given in McClelland (2015) for this specific network model. After training, each network was generally able to activate all units to within 0.20 of their target states for all patterns, indicating that the level of learning was equivalent to that in the work of Lambon Ralph et al. (2007).

Performance, in both intact and damaged networks, was measured by inspecting the name units when visual features were provided as input following the method of Rogers et al. (2004) and Lambon Ralph et al. (2007). Thus, the model’s visual units were clamped to that of the test pattern and activation was circulated through the network for 2 cycles,^8^ after which the visual units were unclamped and activation was recirculated for a further 5 cycles. In a typical intact network, the initial 2 cycles are sufficient to drive the network’s semantic units towards an attractor such that the activation of the visual features due to the semantic units approximates that of the corresponding clamped state. The 5 subsequent cycles then allow the network to settle, ideally to the full pattern (i.e. across all sets of units) associated with the given visual features. Following the 7 cycles of activation circulation, the network’s output was evaluated by examining the activity of the name units. The most active name unit above a threshold of 0.5 was taken as the network’s response. If no name unit was activated above the threshold, “no response” was recorded. This procedure is assumed to be analogous to a participant naming a line-drawing.

Damage to the model was carried out in four ways: by severing a random subset of the connections (by setting their weights to zero); by perturbing connection weights by adding uniformly distributed noise; by removing a random subset of hub units (by setting all output weights from those units to zero); or by scaling weights by a fixed factor between zero and one. In all cases, bias weights (fixed at − 2.0) were left intact, as in Lambon Ralph et al. (2007). For the removal of connections, lesioning was carried out by severing increasing percentages of randomly chosen connection weights (from 2.5*%* to 50*%* at 2.5*%* intervals). For the addition of noise, values were drawn from a uniform distribution centred on zero with an increasing range (from [− 0.05,+ 0.05] to [− 1.00,+ 1.00] at 0.05 intervals) and added to all connection weights (except the bias weights), paralleling the procedure of Lambon Ralph et al. (2007). In the case of removing hub units, each hub unit was considered independently and all output connections from that unit removed with probability *p*, where *p* ranged from 0.00 to 1.00 in intervals of 0.05. For scaling weights, lesions were performed by multiplying the strength of all non-bias weights by a scaling factor (between 0.75 and 0.55 at 0.01 intervals; values outside of this range led to consistent ceiling or floor performance). Each trained network was damaged 10 times for each level of damage, yielding 10 data points for each of 21 levels of damage of each type, for each of the 20 trained networks for each pattern set.

### Results

**Fig. 3:**
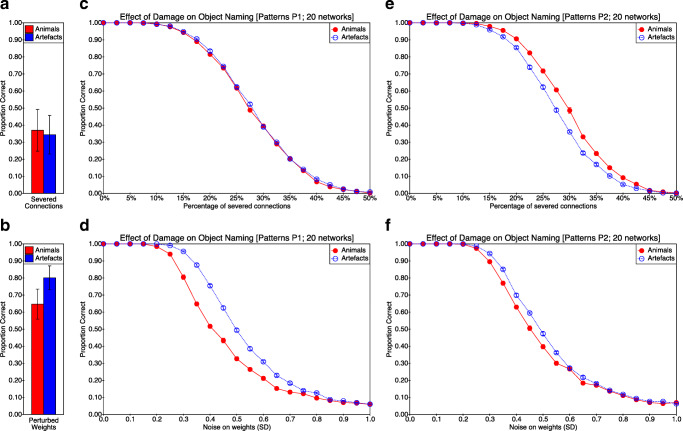
The effect of damage (severing connections or perturbing weights) on naming accuracy for animals and artefacts as reported both in the original simulations and in our attempted replications. The proportion correct for naming for the original simulations as reported by Lambon Ralph et al. (2007) is plotted in **a** when connections are removed and in **b** when noise is added to connection weights. The proportion correct for our simulations trained on P1 is shown in **c** when connections are removed and in **d** when noise is added to connection weights. For our simulations trained on P2, naming accuracy is shown in **e** when connections are removed and in **f** when noise is added to connection weights. The two rows show the models’ performance when weights are removed (top row: panels **a**, **c**, **e**) and when noise is added to connections (bottom row: panels **b**, **d**, **f**). Emphasis in this figure is on the differences or lack thereof between animate and inanimate naming accuracy. Each bar in panes **a** and **b** is based on 7 virtual subjects. Each data point in panels **c**, **d**, **e** and **f** represents mean performance on 10 trials for 20 virtual subjects. Error bars correspond to ± 1 SE

**Fig. 4:**
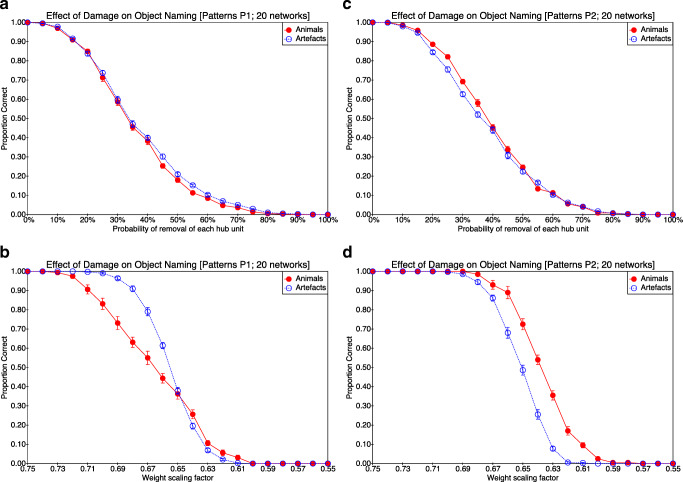
The effect of damage (removing units or scaling weights) on naming accuracy for animals and artefacts for the two pattern sets. The proportion correct when trained on P1 is shown in **a** when units are removed, and in **b** when weights are scaled. For networks trained on P2, the proportion correct for naming accuracy is shown in **c** when units are removed and in **d** when weights are scaled. The two rows show the models’ performance when units are removed (top row: panels **a**, **c**) and when weights are scaled (bottom row: panels **b**, **d**). As in the previous figure, emphasis is on the differences or lack thereof between animate and inanimate naming accuracy, and as in the previous figure, each data point in panels **a**, **b**, **c** and **d** represents mean performance on 10 trials for 20 virtual subjects. Error bars correspond to ± 1 SE

Intact networks name objects at ceiling, but after damage networks show a decline in naming accuracy as a function of the severity of damage, as expected. As can be seen from Figs. 3 and 4, which show naming accuracy for each domain as a function of type of damage and damage severity, this is true for both pattern sets and for all four types of damage. Figure 3 (panels a and b) also shows a summary of the modelling results reported by Lambon Ralph et al. (2007).

#### Qualitative Effects

The relative susceptibility of the network to naming errors in the two domains (animals versus artefacts) differs as a function of the pattern set. At a qualitative level, when trained with pattern set P1, connection removal affects naming accuracy in both domains equally (Fig. 3c; animals = artefacts), while the addition of noise to weights has a more severe effect on naming accuracy for animals than for artefacts (Fig. 3d; animals < artefacts). Removing units is similar to connection removal in that it has similar effects in both domains (Fig. 4a; animals = artefacts), while scaling weights has an effect on naming accuracy that is similar to the addition of noise to weights (at least when accuracy is not severely compromised), being more severe for animals than for artefacts (Fig. 4b; animals < artefacts).

However, a different pattern arises when the network is trained with pattern set P2. In this case, connection removal impacts artefact naming more than animal naming (Fig. 3e; animals > artefacts). The addition of noise following training with pattern set P2 has a similar effect to that seen with pattern set P1 (Fig. 3f; animals < artefacts), though the effect size appears to be smaller. A small but consistent effect in the opposite direction is apparent following unit removal (Fig. 4c; artefacts < animals), an effect that is more apparent following weight scaling (Fig. 4d; artefacts < animals).

#### Quantitative Analyses

The above findings may be quantified by calculating 95% confidence intervals for the area between the curves in each panel of Figs. 3 and 4. Based on the distribution of 20 samples (one for each trained network) for each figure, the 95% confidence interval for the difference in area (artefacts −animals) was calculated for each panel (ignoring the bar charts in panels a and b of Fig. 3). Considering first the severing of connection weights (i.e. Fig. 3 c versus e), the 95% CI for the difference in area when trained on P1 is [− 0.001, + 0.007], while when trained on P2 it is [− 0.022, − 0.016]. In other words, connection severing results in no significant effect of domain when trained on P1 (because the 95% CI includes zero), but a significant effect of domain (with better performance on animal naming) when trained on P2 (because the 95% CI is fully less than zero). With regard to weight perturbation (i.e. Fig. 3 d versus f), both training sets result in a significant advantage for naming artefacts over animals (95% CI for P1: [+ 0.068, + 0.079]; 95% CI for P2: [+ 0.014, + 0.026]), though the effect is larger for training with P1 than with P2 (because the confidence intervals do not overlap). For unit removal (i.e. Fig. 4 a versus c), the difference between curves is small in both cases but significantly positive (i.e. favouring artefacts) when trained on P1 (95% CI: [+ 0.001, + 0.014]) and significantly negative (i.e. favouring animals) when trained on P2 (95% CI: [− 0.019, − 0.006]). The effect is similar when damage is implemented through weight scaling (P1 95% CI: [+ 0.007, + 0.014]; P2 95% CI: [− 0.017, − 0.011]).

#### Is P2 Representative of the Rogers et al. (2004) Template?

The differences in the patterns of breakdown following damage between P1 and P2 are only of concern in as much as P2 is representative of the probabilistic template of Rogers et al. (2004). To assess this, nineteen further pattern sets were generated from the template following the same procedure used to generate P2 but with different random seeds. The network was then trained once on each of the twenty variants of P2. Subsequently, each trained model was damaged following the procedures described above. The mean and standard deviations for the areas between the resultant curves were then calculated for each form of damage. Results are shown in Table 2. As can be seen from the table, the effect of severing connections following training with patterns based on the template generally leads to an animal advantage in naming, while the effect of weight perturbation generally leads to an artefact advantage, both as observed with our initial version of P2. The effects of removal of units and weight scaling are less clear cut, with the mean values obtained from the sample of 20 pattern sets in both cases being within one standard deviation of zero.

**Table 2:** Statistics for the distributions of the area between the artefact and animal naming curves for different forms of damage based on a sample of 20 variants of pattern set P2

Form of damage	Mean (S.D.) area between curves
Weight severing	− 0.012 (0.006)
Weight perturbation	+ 0.039 (0.019)
Removal of units	− 0.006 (0.017)
Scaling of weights	− 0.007 (0.008)

Positive mean values indicate an artefact advantage following damage. Negative mean values indicate an animal advantage following damage

#### Weight Distributions and the “Dimming” of Attractors

A further issue concerns whether the severing of connections can be understood in terms of the “dimming” of attractors, as proposed by Lambon Ralph et al. (2007). This is important because of the contrast with the “distortion” of attractors held to result from perturbation of weights through the addition of noise. As argued in the introduction of this case study, whether dimming is an appropriate analogy in the case of connection severing depends on whether the connections strengths are symmetrically distributed about zero or whether they are predominantly positive. If there are similar numbers of positive and negative weights, then severing connections at random is just as likely to affect inhibitory as it is excitatory connections, and hence is not well described in terms of dimming. Table 3 shows 95% confidence intervals for summary statistics of the weight distributions for networks trained with pattern set P1 and pattern set P2. As can be seen from the table, while there are small differences between the weight distributions as a function of training set, and while these are in some cases statistically significant (given that in some cases corresponding confidence intervals do not overlap), weights resulting from training with both pattern sets are very nearly symmetrically distributed about a mean of zero, and in both cases the proportion of weights that are positive differs only slightly from 50:50 (with a slight majority of weights from visible to hidden and hidden to hidden layers being positive, and a slight majority of weights from hidden to visible being negative). Consequently, severing a random proportion of weights (e.g. in an attempt to model SD) will affect both positive and negative weights and hence will reduce both excitatory and inhibitory influences. It is therefore not well described by analogy with “dimming” of attractors.

**Table 3:** 95% confidence intervals for summary statistics of connection weight distributions in the hub-and-spoke model when trained with P1 and P2

Weight matrix	Minimum	Maximum	Mean	Proportion positive
P1				
Visible to hidden	[− 1.073, − 0.974]	[+ 1.120, + 1.224]	[+ 0.027, + 0.028]	[+ 0.530, + 0.535]
Hidden to hidden	[− 1.011, − 0.929]	[+ 2.733, + 2.845]	[+ 0.038, + 0.039]	[+ 0.513, + 0.520]
Hidden to visible	[− 2.799, − 2.548]	[+ 2.684, + 2.896]	[− 0.037, − 0.036]	[+ 0.431, + 0.435]
P2				
Visible to hidden	[− 1.059, − 0.944]	[+ 1.029, + 1.097]	[+ 0.026, + 0.028]	[+ 0.537, + 0.543]
Hidden to hidden	[− 1.119, − 0.987]	[+ 2.688, + 2.798]	[+ 0.042, + 0.045]	[+ 0.532, + 0.542]
Hidden to visible	[− 2.516, − 2.314]	[+ 2.380, + 2.594]	[− 0.053, − 0.051]	[+ 0.434, + 0.438]

Confidence intervals are based on weight distributions of 20 trained networks. Note that in all cases the networks also include untrainable weights from a bias unit to all units in each layer, with a fixed strength of − 2.0. Bias weights are not subject to damage

### Discussion

#### Summary of Findings

Our attempt at replicating and extending the differential effect reported by Lambon Ralph et al. (2007) of different forms of damage on naming accuracy of animals and artefacts in the hub-and-spoke model has yielded mixed results. With pattern set P1, we obtained essentially the result previously reported—that damaging the model by removing connections impairs naming accuracy of objects in both domains similarly, but that damaging the model by adding noise to weights impairs naming accuracy of animals more than of artefacts, yielding relative preservation of artefact knowledge. This pattern of findings (see Fig. 3 c and d) replicates the difference between SD and HSVE patients reported by Lambon Ralph et al. (2007). We further found (with training set P1) that complete removal of hub units has a similar affect to severing connections (i.e. picture naming accuracy in both domains is affected similarly; see Fig. 4a), while systematically reducing connection weights has a similar effect to adding noise to the weights (i.e. picture naming is more impaired for animals than for artefacts; see Fig. 4b).

Arguably it might have been expected that removal of connections and removal of units within the hub model would yield similar behavioural results, so the first of our additional results may seem unsurprising. In contrast, the second result, concerning the effect of scaling weights, is not so clearly expected, and superficially at least it seems to provide an alternative account of the effect of the HSVE deficit, namely that it is the product of a generalised weakening of connection strengths (or equivalently reduced gain in the activation function). Yet conceptually weight scaling corresponds to representational dimming, a form of representational impairment argued by Rogers et al. (2004) to affect animal and artefact domains equally.

Turning to training with P2, we found a different pattern of results. With this second pattern set, which was constructed from the template given by Rogers et al. (2004), weight noise led to a similar but more mild deficit in naming animals (see Fig. 3f), but removal of connections led to a relative deficit in the naming of artefacts (see Fig. 3e)—contrary to what is typically claimed of SD patients. Removal of hub units and scaling of weights also led to a relative deficit in the naming of artefacts (see Fig. 4c and d). Critically, with three of the four forms of simulated damage, the deficit following training with P2 is reminiscent of the patients of Warrington and McCarthy (1983, 1987), Capitani et al. (2003) and Campanella et al. (2010), rather than HSVE or SD patients.

#### Technical and Methodological Issues

Before considering the results of our simulations in more detail, it is relevant to consider any potential implications of some technical and methodological differences between our studies and those of Lambon Ralph et al. (2007). Firstly, in examining the effects of the different forms of damage, we have plotted naming performance as a function of damage. In contrast, Lambon Ralph et al. (2007) match network performance to patient performance on a separate measure (word-picture matching), in order to control for severity of deficit. Thus, they consider seven levels of connection removal corresponding to each of their seven SD patients and seven levels of weight noise corresponding to each of their seven HSVE patients. This approach of matching network and patient performance is well-justified in that it solves the issue of matching severity across deficits. We note, however, that one patient in each group scored at ceiling on word-picture matching (see Table 1 of Lambon Ralph et al. 2007) and it is unclear how the model might be matched to such patients. In any case, while this is likely to alter the variance in naming performance shown in Fig. 3 (as Lambon Ralph et al. 2007, effectively compare two groups of patients with deficits of varying severity whereas our figure effectively compares homogeneous groups at each level of damage), it should not alter the effect of each form of damage on naming in each domain (i.e. it should not alter the underlying dissociation).

A second technical difference between our studies and those of Lambon Ralph et al. (2007) concerns the level of training of the network prior to damage. The network training parameters and subsequent network performance reported by Lambon Ralph et al. (2007, i.e. 60 patterns trained for 10,000 epochs yielding a maximum error of 0.20) is hard to reconcile with the equivalent details reported in the earlier work of Rogers et al. (2004, where 48 patterns were trained for 400 epochs yielding a maximum error of 0.05). Our approach matched the final error reported by Lambon Ralph et al. (2007). In each of the 48 item pattern sets used here (P1 and P2), this required only 1000 epochs. There is no reason to believe that the model’s behaviour is not robust to these differences in training. First, we used the same procedure to train with P1 and P2, so the difference in results between P1 and P2 cannot be directly attributed to the training procedure. Second, we found qualitatively equivalent results for P2 when the network was trained for 4000 epochs, when the learning rate was doubled and the network was trained for only 500 epochs, and when the learning rate was halved and the network was trained for 2000 epochs. Differences in training between the work reported here and that of Rogers et al. (2004) or Lambon Ralph et al. (2007) therefore do not underlie the difference in post-lesion behaviour of the P1-trained versus P2-trained networks.

#### Three Ways of Interpreting the Results

Our specific results demand greater scrutiny as, superficially at least, they may be interpreted in several ways. Most directly, the fact that the dissociation reported by Lambon Ralph et al. (2007) can be captured when the hub-and-spoke model is trained with pattern set P1 supports the claim that the model can capture the differential effects of SD and HSVE when damaged by removing connections and adding noise to weights respectively. However, the fact that a relative naming deficit for artefacts can arise when the model is trained with pattern set P2 and damaged by removing connections—the reverse pattern to that seen in HSVE patients—suggests that the locus of explanation for the model’s behaviour lies not only with the model architecture (i.e. the hub-and-spoke arrangement) but also with specific aspects of the training set. Moreover, with respect to a theory presented in terms of attractors, it is premature to attribute the model’s behaviour following the two forms of damage to attractor dimming versus attractor distorting (as suggested by Lambon Ralph et al. 2007, but see below for further discussion).

A second way of interpreting the model’s behaviour would be to argue that it supports the existence of a double-dissociation within semantic cognition, with the same model able to produce *either* selective deficits in animal naming (Fig. 3 d, f or b) *or* selective deficits in artefact naming, as a function of the pattern set (Fig. 3 e, c or d). As discussed previously, both types of selective deficit have been observed in patient studies. This interpretation is consistent with arguments in the neuropsychological literature that attribute different category-specific deficits (or at least a selective deficit in artefact naming, which appears to be less common than a selective deficit in animal naming) to pre-morbid differences in the cognitive system that might result from individual domain-specific expertise (e.g. Jefferies et al. 2011), though it should be stressed that patients who show this reverse category-specific deficit typically have lesions affecting posterior regions of the left temporal lobe (Campanella et al. 2010), while the damage of those showing the more standard category-specific deficit is typically localised to more anterior regions of the temporal lobes (see footnote 2). Nevertheless, applying the interpretation to the hub-and-spoke model without substantiating pre-morbid differences would compromise the model’s falsifiability. While falsifiability is not necessarily the be-all and end-all of scientific theorising, additional theoretical assumptions require additional empirically validated predictions (in this case, concerning pre-morbid differences in experience) if a research programme is to avoid scientific degeneracy (Lakatos^1970^).

A third way of interpreting our results is that the pattern sets differ in the relative difficulty of animal naming and artefact naming. For example, suppose that as hypothesised, animal naming is more susceptible to noise than artefact naming, but that animal naming is in general easier or more robust than artefact naming in P2, but not in P1 (or equivalently, artefact naming is easier than animal naming in P1, but not in P2). In other words, and taking the severing of connections as providing a kind of baseline (Fig. 3 c and e), the effect of weight noise relative to that baseline can be seen in both pattern sets to be effectively a shifting of the curve representing animal naming accuracy to the left. Panel d of Fig. 3 may be understood as corresponding to panel c but with the curve representing animal naming shifted to the left. Panel f can be derived from panel e by the same translation. While this pair of assumptions could account for the pattern of results in Fig. 3, this interpretation requires some way of quantifying “robustness to damage” for domain-specific naming in abstract terms (i.e. without reference to the type of damage).

#### Robustness to Damage and the Nature of Attractors

One possible approach to quantifying robustness to damage across domains is in terms of the average error in the naming task (e.g. as measured by the Euclidean difference between the target output vector and the actual output vector, or between the name units in each vector) prior to damage. Table 4 presents this error for each domain and each training set. For both P1 and P2, the average pattern error (i.e. over all 216 interface units) is greater for animals than artefacts, but if one restricts attention to just the name units then the pattern is reversed—the average error is less on animals than artefacts. While the latter could be taken to suggest that knowledge of animal names should be more robust than knowledge or artefact names, regardless of training set, the former suggests the opposite. Focussing more specifically on the differences due to training set, the pattern error figures suggest animal knowledge should be least robust when the model is trained with P2, yet inspection of Fig. 3 suggests the reverse. Alternatively, the naming error figures suggest artefact knowledge should be least robust when the model is trained with P1, which again is not borne out by Fig. 3. Hence, there is no support for the proposal that animal knowledge is, on the whole, relatively stronger than artefact knowledge following training with P2 compared with training with P1.

**Table 4:** Mean Euclidean distance between output and target patterns (left columns) and between output and target name units (right columns)

	Pattern error		Name error	
	Animals	Artefacts	Animals	Artefacts
P1	0.657	0.642	0.202	0.223
P2	0.671	0.642	0.207	0.212

Distances are averaged over the 20 separately trained networks for each pattern set used to generate Fig. 3. Note that pattern error and name error are not directly comparable, as patterns comprise 216 units while names comprise 40 units

An alternative approach to quantifying robustness is based on the density of attractors within the vector space defined by hidden unit activation. If, in a portion of that vector space, the distance between attractors is relatively large (i.e. attractors are sparsely arranged) then those attractors should be relatively more robust to damage compared with attractors from within a more densely populated region of the space. Indeed, Rogers et al. (2004) explicitly appeal to attractor sparsity/density as an explanatory concept, arguing that, in their model, “artifact representations are more sparsely distributed across a broader region of the space” (p. 231). Their appeal to the differential distributional properties of attractors as a function of semantic domain is motivated by elicitation studies of feature norms (primarily Garrard et al. 2001; see also Dilkina and Lambon Ralph 2013; Devereux et al. 2014). While Rogers et al. do not quantify attractor density, their claim—that animal subspace is more densely populated than artefact subspace—is supported by our analysis of the attractors resulting from both pattern set P1 and pattern set P2. (See Table 5 and discussion below.)

**Table 5:** Mean Euclidean pairwise distance between attractor states within each domain in the hub-and-spoke model for each pattern set

	Animals	Artefacts
P1	4.0806	4.1984
P2	4.2428	4.3505

Distances are averaged over the 20 separately trained networks for each pattern set used to generate Fig. 3

With robustness equated with attractor sparcity, and with the third of the above interpretations in mind, one might hypothesise that animal naming is slightly better preserved in P2 than in P1 (or artefact naming is slightly better preserved in P1 than in P2) because animal attractors are more sparsely arranged (in comparison with artefact attractors) in P2 than in P1 (or artefact attractors are more sparsely arranged, in comparison with animal attractors, in P1 than in P2). By way of exploring this possibility, Table 5 shows the mean Euclidean distance between all pairs of animal attractors and between all pairs of artefact attractors for each pattern set. Lower values indicate denser packing of attractors within attractor space, since the distance between them is smaller. The table (and subsequent statistical analysis) shows that animal attractors are more densely arranged than artefact attractors for both P1 and P2, and that attractors resulting from P2 are sparser than those arising from P1, but there is no interaction between these factors (main effect of domain: *F*(1,38) = 21.414, *p* < .001; main effect of pattern set: *F*(1,38) = 57.277, *p* < .001; interaction of domain and pattern set: *F*(1,38) = 0.043, *n*.*s*.). This analysis therefore argues against the third possible interpretation of our results: for both P1 and P2, animals are more densely organised than artefacts following training, but both domains are similarly more sparsely arranged following training with P2 than with P1, suggesting that both should be similarly more robust following training with P2 than with P1.

There is in fact another systematic difference between P1 and P2 alluded to in the “Characteristics of Pattern Sets” section, namely that animal representations in P1 have greater norms (i.e. more features present per pattern) than in P2, with approximately 42 features per animal pattern in P1 but just 36 per animal pattern in P2. This is not the case for artefact representations, which differ by only 1 to 2 features per pattern between P1 and P2. In a series of investigations with further pattern sets, as described in the Supplementary Materials, we found that the behaviour of the model when trained with pattern set P1 could be reproduced by a pattern set based on P2 if the norms of animal vectors in that pattern set were increased to levels similar to those in P1. Rogers et al. (2004) argue that animal representations are both richer (i.e. have more features present) and more confusable than artefact representation. Our simulations show that these properties have opposite consequences for robustness of the representations following connection severing. Capturing the generalised deficit of SD via connection severing within the hub-and-spoke model requires trading off these properties across the domains, where in the model increased richness of animal representations compared with artefact representations is balanced by increased confusability between animal representations compared with artefact representations.

#### Dimming and Distortion Revisted

The concept of attractor density also needs to be reconciled with the explanatory role of “dimming” and “distortion” as introduced by Lambon Ralph et al. (2007). Recall that these concepts were invoked to explain differences between SD and HSVE at the level of attractors. Removal of connections was held to result in dimming while the addition of noise to weights was held to result in distortion. Dimming was held to have similar effects on the internal representations of animals and artefacts (and so affect naming of both similarly). In contrast, distortion was held to have disproportionately detrimental effects on the internal representations of animals, where in the undamaged system animal attractors are more densely packed.

At an abstract level, this explanation for the difference between SD and HSVE may seem plausible. The critical issue is whether, as noted earlier, connection removal and weight perturbation can reasonably be characterised as resulting in, respectively, dimming and distortion of attractors. The evidence discussed above concerning the distribution of weights in the trained networks casts doubt on this characterisation, but a further query follows from our consideration of the effects of weight scaling. The explanatory claim of attractor dimming is that attractors will be more confusable if they are shallower, and because of domain-dependent differences in the packing of attractor space, this will impair animal naming more than artefact naming. Lambon Ralph et al. (2007) considered only two forms of damage, but of the four forms of damage discussed here, that which most closely affects depth of attractors is not connection severing but weight scaling—systematically reducing weights by a fixed factor would, a priori, seem more likely to produce shallow attractors than randomly removing a subset of connections. Yet, as shown in Fig. 4 b and d, scaling weights down impairs artefact naming more than object naming—the opposite dissociation to that which is argued by Lambon Ralph et al. (2007) to reflect attractor dimming.

#### Conclusion

Regardless of the differences in the network’s behaviour following damage when trained with P1 versus P2, it is clear that removal of connection weights and addition of weight noise within the hub-and-spoke model can affect the naming of objects and the naming of artefacts differently, as can removal of hub units and scaling of connection weights. In other words, and in contrast to the assumption often implicit in connectionist modelling of neuropsychological deficits (such as the majority of work cited in the introduction), different forms of damage to identical loci within the hub-and-spoke model may result in different deficits. Thus, it is clear that the hub-and-spoke model can show category-specific semantic impairments, and that qualitatively different impairments may arise following different types of damage.

Equally, the precise form of the impairment depends not just on the type of damage, but also on specific aspects of the training set. Seemingly minor differences between training sets can drive substantial differences in network behaviour following lesioning, so much so that the headline result, of no category-specific deficit following the severing of connections but a specific impairment of animal naming following the addition of noise to connection weights, may be reversed, yielding a specific impairment of artefact naming following the severing of connections (albeit with a residual mild animal naming deficit following perturbation of weights through the addition of noise). Indeed, the headline result of Lambon Ralph et al. (2007) does not follow when the hub-and-spoke model is trained with patterns generated from the template presented by Rogers et al. (2004), despite the fact that both the patterns of Rogers et al. (2004) and of Lambon Ralph et al. (2007) are based on the same source—the feature norms of Garrard et al. (2001).

Our results also raise questions about the robustness of the original simulation results reported by Rogers et al. (2004). More specifically, are the original results a product of the specific training set used in that work, or would they hold with the training set used in the later work of Lambon Ralph et al. (2007)? Given that our focus in this paper is on the effect of different forms of simulated damage and not specifically the hub-and-spoke model, we have made no attempt to replicate all simulations of Rogers et al. (2004) with the current pattern sets.

## Case Study 2: Lesioning a Recurrent Network Model of the Control of Routine Sequential Action

### Rationale

The previous simulation study demonstrated that several standard ways of implementing neural damage within a connectionist model may result in post-lesion behavioural differences, but these differences may also be dependent upon seemingly irrelevant aspects of the training set. This second case study further explores this issue using a different domain and a different class of model. More specifically, this case study involves exploring the effects of different forms of damage on the behaviour of the simple recurrent network (SRN; Elman 1990) model of the control of (routine) sequential action selection proposed by Botvinick and Plaut (2004), with the aim of understanding whether post-lesion behavioural differences in connectionist models are potentially general properties of connectionist models and their training sets, or dependent upon the specific distinction between animals and artefacts within the semantic cognition domain.

**Fig. 5:**
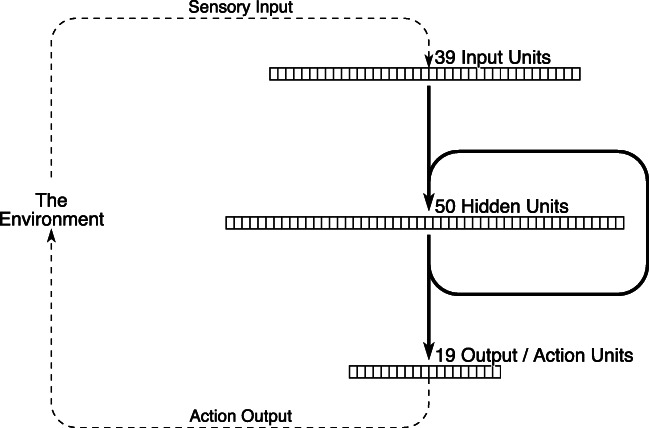
The architecture of the simple recurrent network model of sequential action selection of Botvinick and Plaut (2004)

The SRN model of sequential action selection, whose architecture is shown in Fig. 5, interacts with a simplified environment via a simulated eye and a simulated hand, taking as input a featural representation of the currently fixated and currently held objects, and producing as output a discrete representation of an action (e.g. “put down” or “fixate spoon”). The environment includes a set of (simulated) objects relevant to beverage-preparation, such as a tea bag, sugar bowl and coffee sachet. Botvinick and Plaut (2004) trained the SRN using backpropagation through time with six different extended goal-directed action sequences—four ways of preparing coffee and two ways of preparing tea—as well as a set of one-step background actions (e.g. if the hand is free and the eye is fixated on the spoon then a possible action is to “pick up”). Following training, the model was able to reproduce each of the six extended action sequences.

The model is relevant here because of two additional simulation studies reported by Botvinick and Plaut (2004). Firstly, when the representation of context within the model was disturbed (by the addition on each processing step of normally distributed random noise, with a mean of zero and a relatively low variance, to the activation of context units, i.e. the hidden units on the preceding time step), the model produced a range of errors reminiscent of everyday slips and lapses in action. Errors included occasional omissions and perseverations (i.e. inappropriate repetitions) of action subsequences. These were held to mirror the occasional slips and lapses that often occur in routine or everyday behaviour (Norman 1981; Reason 1979, 1984). Secondly, more extreme disturbance of the context units (modelled by addition of noise with greater variance to the hidden units) led the model to produce errors in action selection similar to those produced by neurological patients with action disorganisation syndrome (ADS: Schwartz et al.1998a, 1998b; Humphreys and Forde 1998; see also Schwartz et al. 1991). These patients have trouble in producing organised sequences of actions during goal-directed behaviour, often leaving out actions, adding inappropriate actions or repeating actions unnecessarily. Specifically, Botvinick and Plaut (2004) argued that:

1. With mild damage, errors in the network’s action selection occur at subtask boundaries (e.g. after adding sugar to the coffee but before adding cream), rather than within subtasks (e.g. during the “adding sugar” sub-routine), despite that fact that the network was not trained with explicit sub-routines (e.g. for adding sugar or adding cream).
2. Also at mild levels of damage, errors in the network’s action selection generally take the form of whole subtasks intruding in a sequence of actions (including perseveratively) or being omitted from a sequence, rather than single action errors or within-subtask disorganisation.
3. When actions are divided into “crux” and “non-crux” actions, with the former resulting in the notional fulfilment of a goal (e.g. the act of emptying a spoonful of sugar into a coffee cup, which achieves the goal of sweetening the beverage) and the latter consisting of preparatory and clean-up actions (e.g. scooping sugar into the spoon from the sugar bowl in preparation for adding sugar to the beverage, or putting the spoon down after the sugar has been added), greater levels of noise/more severe damage results in a higher proportion of non-crux errors than crux errors, as well as an increased proportion of so-called independent actions (i.e. actions that do not support a temporally adjacent crux action).
4. With more severe levels of damage, the most common type of error is an omission error (where an action or subtask is omitted). Sequence errors (subtask perseverations, action anticipations etc.) are the second most common type. Other types of errors (e.g. object substitution errors, such as attempting to stir with a coffee packet, and tool omission errors, such as pouring the sugar from the sugar bowl instead of scooping it with a spoon) also occur but with much lower frequency.

The purpose of the set of simulations described in this section is to determine whether these properties are a function of the specific type of damage considered by Botvinick and Plaut (2004) or whether they might be more general properties of the SRN model following breakdown. In other words, as in the previous case study, we wish to investigate if the network’s behaviour after lesioning is a function of the way in which it is lesioned.

Critically, like the hub-and-spoke model considered previously, which settles given fixed input, the model’s behaviour depends upon attractor dynamics that develops through learning. However, the attractors are different in character because with the Botvinick and Plaut (2004) SRN each step of processing results in an action which changes the environment, and hence the input to the network on the subsequent step. Thus, the model does not settle with fixed input to an attractor state, but over time produces a sequence of outputs. Notwithstanding, it remains appropriate to describe the network in terms of attractor dynamics as, when given the same input but different initial activation in the hidden units, the network tends to produce the same sequence of outputs (subject to variability in the training set). Moreover, examination of the hidden unit activations reveals that, within each action sequence known to the network, the variability between hidden unit activation vectors decreases with each step in the sequence. That is, the difference between the hidden unit activation vectors for the trained model on, e.g. two instances of preparing tea with sugar from a packet on step *N* + 1 is less than that on step *N*, implying that the path taken by the model through the multi-dimensional hidden unit vector space can be understood as a kind of attractor (albeit a “path attractor” rather than a “point attractor”).

Given the difference in the character of attractors between the recurrent attractor network considered earlier and the Botvinick and Plaut (2004) SRN—namely that the former are points in state-space and the latter are paths—it is not clear that the earlier results concerning differential effects of different forms of damage will hold here. Moreover, if different forms of damage yield equivalent deficits, then we may conclude that the factors driving the sensitivity to damage type within the recurrent network used to implement the hub-and-spoke model by Lambon Ralph et al. (2007) are either not present in the SRN used to model ADS by Botvinick and Plaut (2004) or not relevant to performance—normal or impaired—of the specific sequential tasks (coffee preparation and tea preparation) modelled by the SRN.

While the account of ADS given by Botvinick and Plaut (2004) remains contentious (see Cooper and Shallice 2006, for arguments against the account, Botvinick and Plaut, 2006, for a rebuttal, and Cooper et al. 2014, for a potential rapprochement), there are three additional reasons for considering the effects of different forms of damage within the model under the current research programme. Firstly, the form of damage considered by the original simulations of ADS involves the addition of noise to context unit activation values. That is, it does not involve any of the four forms of damage considered earlier in this paper (i.e. perturbing weights through the addition of random noise, removing connection weights, removing hidden units or scaling weights). In fact, processing within SRN models lends itself to this fifth form of damage, which is less appropriate for simulating damage in a recurrent attractor network, where noise added to hidden units on each cycle would prevent the network from settling. A key question is therefore whether all five forms of damage are (dys-)functionally equivalent within the model, or whether ADS-like behaviour is dependent on the specific form of damage investigated by Botvinick and Plaut (2004).

Secondly, the patterns on which the network was trained—coffee-making and tea-making sequences—have different characteristics, with the former including variable subsequence order (sugar and cream can be added in either order when preparing coffee) but the latter including only variability due to input from the simulated environment (sugar may be added from a packet or a bowl, depending on which sugar source the simulated eye happens to fixate on). These different types of sequence can be regarded as representing an indirect analogue of the different domains of object (living things and artefacts) considered in the earlier simulations. More specifically, the contrast between coffee making and tea making allows for the possibility that different tasks will be prone to different types of error, and that such differences could interact with the way in which neural damage is implemented.

Finally, the results might help to address outstanding questions concerning the breakdown of action selection following neurological damage. Action disorganisation syndrome has been reported in patients with frontal cortical damage caused by closed head injury (Schwartz et al. 1998b), stroke (Buxbaum et al. 1998; Humphreys and Forde 1998; Schwartz et al.1998a) and carbon monoxide poisoning (Humphreys and Forde 1998), amongst others. It appears that the form of the behavioural impairment (i.e. ADS) is dependent upon the site of the neural damage (broadly speaking, frontal cortex^9^), but is not sensitive to the origin or cause of the neural damage.^10^ If the model’s behaviour proves to be insensitive to the way in which damage is implemented, then this may potentially be taken to support the position that the different approaches to modelling damage can be equated with different types of damage at the neural level. More critically, it may be taken as support for the utility of an information-processing level of description that abstracts from neural implementation but captures both the empirical regularities of interest (i.e. the behaviour of ADS patients) and the independence of those regularities from the precise form of damage at the neural/implementation level that gives rise to them.

### Method and Results

Botvinick and Plaut (2004) describe their model in considerable detail, and in previous work a fully independent reimplementation of the model was produced on the basis of their published description (Cooper and Shallice 2006). This reimplementation, which has previously been shown to reproduce the four key findings described above when the activations of context units were perturbed, served as the basis for the simulations reported here. The reimplementation was trained for 20,000 epochs on the six extended multi-step action sequences (two ways of preparing tea, each consisting of 20 steps, and four ways of preparing coffee, each consisting of 37 steps) and a larger set of (250+) single-step actions as described by Botvinick and Plaut (2004). This process was repeated 11 times with networks initialised with different random weights prior to training, to give a sample of 12 trained networks. We verified that the trained but undamaged reimplemented networks could reproduce all six of the training sequences (by successfully reproducing Table 4 of Botvinick and Plaut 2004). Four sets of five simulation studies (i.e. twenty in total) were then run to address the key findings noted above.

**Fig. 6:**
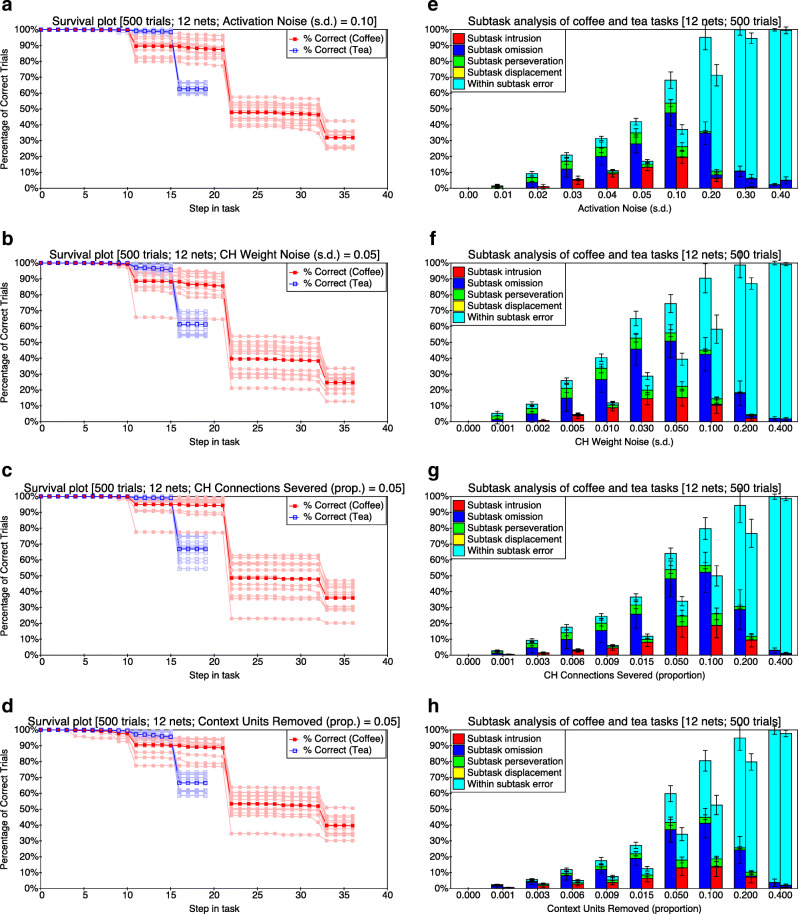
Results of damaging the SRN model of routine action selection of Botvinick and Plaut (2004). Left column: Survival plots for the moderately damaged network preparing coffee/tea, showing how errors tend to occur at specific steps in the action sequence. Right column: Subtask errors as a function of damage level for coffee preparation (left bars) and tea preparation (right bars), showing how mild-to-moderate damage results primarily in subtask omission, while more severe damage results primarily in within-subtask errors. For the top-most row, **a** and **e**, damage was effected by perturbing the activations of hidden units. For the second row, **b** and **f**, damage was effected by perturbing connection weights. For the third row, **c** and **g**, damage was effected by severing connections, and for the lowest row, **d** and **h**, damage was effected by removing context units. For survival plots, darker lines represent means over 12 separately trained networks, while lighter lines represent the results from individual networks. For the subtask analyses, bars represent means for the same 12 networks, with error bars showing one standard deviation

Firstly, the 12 trained models were run 500 times each for each of the two tasks (preparing coffee and preparing tea) with zero-mean normally distributed random noise (with standard deviation of 0.10) added to the context unit activations on each processing step (paralleling simulation 2 of Botvinick and Plaut 2004). Survival plots, which show the percentage of trials correct up to each step of each task, were then produced (see Figure 8 of Botvinick and Plaut 2004). As in the original model, errors tended to occur at subtask boundaries (steps 10/11, 21/22 and 32/33 when making coffee, which correspond to completion of adding coffee granules, sugar and cream, and steps 10/11 and 15/16 when making tea, which correspond to the completion of pouring in and stirring the sugar; see Fig. 6a). The simulation was repeated with damage implemented instead by adding zero-mean normally distributed random noise (with standard deviation of 0.05) to the recurrent connection weights (i.e. the context unit to hidden unit weights) after training but prior to testing. Survival plots were produced for the 500 instances of the 12 perturbed networks on each of the two tasks (see Fig. 6b). Again, errors tended to occur at subtask boundaries. The simulation was then performed a third time with damage implemented through severing 5% of the recurrent connections (i.e. by setting, with probability of 0.05, the strength of each of those weights to zero). Again, survival plots were produced for the 500 instances of the 12 damaged networks on each of the two tasks (see Fig. 6c), and again errors tended to occur at subtask boundaries. A further simulation was performed with damage implemented through removal of 5% of the context units (or more precisely, with 5% probability for each context unit, that all connections from the unit were set to zero). Survival plots were produced for 500 instances of the 12 damaged networks on each of the two tasks (see Fig. 6d), and yet again errors tended to occur at subtask boundaries. Finally, the simulation was repeated with damage implemented through scaling of weights. That is, all non-bias weights were multiplied by a fixed factor less than one. Survival plots (not shown) were again produced for the 500 instances of the 12 damaged networks on each of the two tasks. Failure specifically at subtask boundaries was again observed, though the degree of scaling leading to failure varied with task. Thus, failure occurred in coffee making only when the scaling factor was less than 0.88 and in tea making only when the scaling factor was less than 0.49.

A second set of five simulations explored the types of error (e.g. subtask intrusion versus subtask omission) resulting from different levels of damage. Firstly, the networks were damaged with context activation noise as in the original work of Botvinick and Plaut (2004). Analogous simulations were then performed with weight noise, connection severing, context unit removal and weight scaling. In each case, errors were classified as subtask errors (intrusions, omissions, perseverations and displacement) or within-subtask errors. In all cases apart from weight scaling, and as in the original simulations of Botvinick and Plaut (2004), mild-to-moderate levels of damage led to an increase in the number of subtask errors, while more extreme levels of damage led to a preponderance of within-subtask errors (see Fig. 6e, f, g and h). Of particular note is the observation that, with mild-to-moderate damage, coffee making is particularly prone to subtask omission errors, while tea making is particularly prone to subtask intrusion errors. The same qualitative pattern holds across all types of damage with the exception of weight scaling. Severe weight scaling (when the scaling factor is less than about 0.2) reduces all activations to 0.5 and is of no theoretical interest. For less extreme values, weight scaling results in no errors if the scaling factor is above a task-specific threshold (0.88 for coffee making and 0.49 for tea making) and subtask omission errors if the scaling factor is below that threshold.

The third set of simulations explored how level of damage affected the proportion of independent actions and the ratio of crux to non-crux errors across both tasks. Networks were again tested with a range of levels of each type of damage. The proportion of actions that were independent (i.e. that did not cohere within a subtask) was plotted as a function of level of damage, as was the proportion of actions that were crux and non-crux errors (see Supplementary Materials Figure 14). Again with the exception of weight scaling, each type of damage produced the same pattern of results—higher levels of damage resulted in higher proportions of independent actions, with most errors being non-crux errors. Furthermore in all three cases, the proportions were similar for both tasks (coffee preparation and tea preparation) and the ratio of non-crux to crux errors was (to a first approximation) constant.

In the final set of simulations, the model’s errors were analysed by type (omission versus sequence versus other). For each form of damage (apart from weight scaling), low levels of damage resulted in similar rates of omission and sequence errors, and very low rates of other errors, while high levels of damage resulted in a preponderance of omission errors (at least with coffee preparation; see Supplementary Materials Figure 15). While the precise pattern of results varied by task (coffee preparation versus tea preparation), it did not vary across four of the five types of damage. For the fifth type of damage (weight scaling), all errors were omission errors—no sequence or other errors were observed in either task.

### Discussion

The above simulations fully replicate the results of Botvinick and Plaut (2004), both in terms of intact functioning of the network and impaired functioning following the addition of noise to context unit activations (as previously reported in Cooper and Shallice 2006). They also demonstrate that this behaviour is not dependent on the network’s initial weights, as the results hold over 12 independently trained instances of the network. In addition, however, the network’s impaired behaviour has been shown to be task sensitive, in that there are clear differences between the types of error produced by the damaged network when attempting the coffee task versus the tea task (as shown in Fig. 6e, f, g and h). Moreover, precisely the same patterns of results were found to hold when the network was damaged through the addition of noise to the network’s weights, or when a proportion of the network’s connections were severed, or when a proportion of the network’s context units were removed. The only form of damage that did not result in this pattern of results was weight scaling. Thus, while the damaged network’s behaviour is task sensitive, it is largely insensitive to the type of damage. Moreover, with the exception of weight scaling, there is no qualitative interaction between task and type of damage. This builds on the results of the earlier case study by suggesting that whatever drives the differential response to different types of damage in the hub-and-spoke model of semantic cognition does not operate within the Botvinick and Plaut (2004) network, in spite of our consideration of five distinct forms of damage and two tasks with different characteristics.

Our results are arguably not surprising in the context of Botvinick and Plaut’s (2004) rationale for implementing damage via the addition of noise to context units. Specifically, they argue that “the most direct way to compromise the mechanisms that support sequencing is to disrupt the information carried by the recurrent connections within the hidden layer. The disruption of internal representations can be viewed as corresponding, in terms of its consequences, to basic etiological factors underlying both slips of action and ADS. Studies of slips have emphasised that such errors tend to occur during periods of distraction, during which there is cross talk from task-irrelevant cognitive activity (Reason, 1990). The addition of noise to [internal representations of task context] can thus be understood as a functional correlate of mental distraction. More severe levels of noise can be interpreted as representing the effects of direct neural damage in ADS” (Botvinick and Plaut 2004, p. 401). The authors further note that a variety of methods have been used to induce disruption in connectionist networks (as discussed throughout this paper), with the implication being that any form of damage that disrupts the influence of context representations in a non-systematic way should be (dys-)functionally equivalent, and hence that any or all of these methods might have been used in their simulations of action slips and lapses and of action disorganisation syndrome. This position reflects the level of abstraction at which the model operates. That is, the network can be understood not just as a model of neural function (for which it is arguably a poor model, given the lack of any clear relation between units in the model and neural elements, or between the model’s learning algorithm and that of the brain), but also as an information-processing model operating at the level of transforming abstract representations of the environment, task context, and atomic actions. This more abstract level is, we contend, appropriate for understanding cognitive-level properties of connectionist/parallel distributed processing networks, and our simulations support this position.

This second case study also shows that arguments about type of damage may to some extent be generalised from the forms of damage considered in the earlier simulation study (i.e. severing connections, perturbing the weights of those connections, removing units and scaling weights) to other forms of damage (i.e. perturbing activation signals). Four of the five forms of damage have been shown to be equivalent for the SRN model, at least with respect to the specific tasks considered, with the four equivalent forms of damage yielding qualitatively distinct error profiles on the two tasks (for coffee preparation and tea preparation), but qualitatively equivalent error profiles within each task. The only exception to this concerns scaling of weights, which, as noted, was only detrimental to the model’s performance when scaling was beyond a task-specific threshold, when it led to the SRN omitting subtasks.

It is worth reconsidering the specifics of the tasks given this pattern of breakdown. The key difference in behaviour on the tasks following damage, shown across Fig. 6 panels e to h, is that at low to moderate levels of damage subtask omission errors are the most common error type during coffee preparation, but subtask intrusion errors are the most common error type during tea preparation. It has previously been argued that errors in the SRN model arise when similar task contexts (as represented by the activation of context units) are conflated (Botvinick and Plaut 2002). Cooper and Shallice (2006) argue that all of the model’s errors are effectively “capture” errors (Reason 1979), where behaviour on one task is “captured” by another similar task. Thus, omissions occur in coffee making because action is captured by the related task of tea making (which does not involve the addition of cream but is otherwise similar, and hence the creaming subtask is omitted), while subtask intrusion errors occur in tea making because action is captured by coffee making (which does involve the addition of cream but is otherwise similar, and hence this subtask intrudes). Our simulation results suggest that the factors that determine whether one task may capture, or be captured by, another are not dependent on whether damage is implemented through perturbation of context activation, severing of connection weights, perturbation of those weights, or removal of context units.

A final issue relating to this set of simulations concerns the role of aetiology as opposed to lesion site in ADS. Recall that, despite some reported differences in lesion site in SD and HSVE, the difference in aetiology, rather than lesion location, was suggested by Lambon Ralph et al. (2007) to underlie the behavioural differences between those two patient groups in tests of semantic knowledge. One interpretation of the simulations presented in this section is that they provide insight into why behavioural differences do not occur in ADS, despite differences in aetiology. That insight is that four of the five forms of damage implemented in the simulations have qualitatively equivalent effects on the representation of task context. That is, within the SRN model, different tasks (with different characteristics and arguably with different complexity) do not appear to give rise to task contexts that are differentially sensitive to different types of damage (unlike the recurrently connected units of the hub model, where different classes of stimulus, with different numbers of associated features, do).

Extrapolating from this, and considering the results of the first case study, one might speculate that if the number of features contributing to inputs for the different tasks were different (e.g. if one were to borrow the representational scheme from the hub-and-spoke model for the representation of fixated/held objects and then compare tasks involving actions applied to animals as opposed to actions applied to artefacts) then one would expect different forms of damage to result in task-related differences in error profiles.

## General Discussion

Connectionist modelling has proven to be highly productive in allowing the specification of concrete accounts of cognitive processing and in linking those accounts with behavioural findings from studies of both neurologically healthy participants and patients with neurological impairment. In this article, we have explored the mechanisms underlying the modelling of neuropsychological impairments by considering the behavioural consequences of different approaches to implementing neural damage within two connectionist models. As noted in the introduction, this was motivated by a perceived tension between the results of Plaut and Shallice (1993a and of others), who found no qualitative difference between the effects of different approaches to simulating damage, and the claim of Lambon Ralph et al. (2007), that different forms of generalised damage within a connectionist model of semantic cognition give rise to different semantic impairments—impairments that are associated with different aetiologies.

Our case studies have yielded a mixed picture, with damage-dependent differences in simulated behaviour arising in one case (the model of Lambon Ralph et al. 2007), but not another (the model of Botvinick and Plaut 2004). We further found that the behavioural consequences of simulated damage are more sensitive to training pattern structure than might be expected: in the model of Lambon Ralph et al. (2007) differences in training patterns yielded behavioural differences following different forms of damage. Moreover, these differences were substantive. When, for example, the hub-and-spoke model was damaged through the severing of connections, training with one pattern set led the damaged model to produce the behavioural pattern of semantic dementia, while training with another closely related set led the model to produce a relative deficit in artefact knowledge—a behavioural pattern that has been associated with left posterior temporal tumour patients (Campanella et al. 2010). In this general discussion, we consider the consequences of these findings both for the specific models and for connectionist cognitive neuropsychology more generally.

### Connectionist Modelling and Credit Assignment

It is well known that the behaviour of connectionist networks is sensitive to the exemplars on which they are trained. Guest and Love (2017) provide a particularly stark demonstration of this, by showing that even untrained feed-forward networks (i.e. networks with random weights initialised as they would be prior to training) preserve some level of the similarity structure of their inputs, and this holds even for deep networks of up to eight layers. What is perhaps less well appreciated is that a consequence of this sensitivity is that one cannot, without a good deal of additional analysis, uniquely attribute a model’s success to either its architecture or its training set.

In other words, in successful connectionist modelling there is a credit assignment problem: Is behaviour the result of the architecture or the training set, or some interaction between the two? This problem is always present in connectionist modelling, but is magnified when we are also considering the effect of (different forms of) damage. As discussed below, there is a simple methodological ploy to tackle the problem—varying one or more components to determine how the model’s behaviour depends on each—but the more common approach is to avoid discussion of credit assignment altogether. The default assumption is typically that modelled behaviour is the product of the architecture, with the training set and implementation of damage being considered as secondary.

There are exceptions. In the study of semantic/conceptual knowledge of Tyler et al. (2000), for example, correlational structure between items that comprise the training set (or more generally, the featural descriptions of the objects to which the network, and by extension, human participants, are exposed) is explicitly the critical theoretical assumption that is held to drive the simulation results. Yet even there the authors consider only one architecture (a standard feed-forward auto-associative network) and one approach to modelling damage (lesioning a proportion of connections).

Within the hub-and-spoke account of semantic cognition the picture is more complex. Here it is clear that Rogers et al. (2004) view the simulation results as the product of all three—the architecture, the training set (or at least its statistical properties), and the implementation of damage. Yet, with the exception of the simulations of Lambon Ralph et al. (2007, which we have already critiqued), systematic variation of each component in isolation has not previously been reported. Thus, a difficulty for the hub-and-spoke model is that at present we do not know where explanatory credit should be assigned—to the training set, to the architecture, or to the implementation of damage. This difficulty remains even if we are only considering the undamaged model and its account of non-impaired semantic cognition.

Similar arguments can be extended to the model of sequential action selection of Botvinick and Plaut (2004). Behaviour of the undamaged network is held to reflect both the recurrent network architecture and the training set, while behaviour following damage was modelled by activation noise in the context layer (which in turn was held to reflect degradation of internal representations), yet in the original work none of the three components was shown to be necessary. In this case, however, the authors’ arguments essentially concerned the sufficiency of the proposed architecture, i.e. they were arguing that a simple recurrent network was capable of generating both normal and impaired sequential action selection. As such, independent variation of the separate components was not essential to support their claims.

At the same time, a key finding of Botvinick and Plaut (2004) was that, when damaged, their SRN model produced a preponderance of omission errors in the routine sequential task of coffee preparation. This parallels the tendency of some frontal patients to make particularly high rates of omission errors. In the simulations reported here, we found that the tendency of the trained model to produce omission errors is task specific—subtask omission errors are common when making coffee but subtask *intrusion* errors dominate when the task is to make tea. The reason is the structure of the training set: the model is trained on only two tasks. If coffee making is the intended task but behaviour is captured by tea making then omission errors are likely. If tea making is the intended task and behaviour is captured by coffee making then intrusion errors are likely. In order to safely conclude that the model provides a viable explanation for the high frequency of omission errors, it is therefore necessary to train the model with many more tasks, presumably distributed in accordance with the statistics of everyday experience. Our simulations suggest, for example, that if the model were trained on more task sequences, and hence more tasks could potentially intrude on the intended task, then intrusion errors, rather than omission errors, would dominate.

Closely related to the role of the training set in a network’s behaviour is the role of training parameters. We have not here systematically reported studies varying training parameters, but in additional simulations with both the hub-and-spoke model and the SRN model of sequential action selection, we found no qualitative effect on behaviour following damage of varying the number of training epochs or the learning rate, provided that the network adequately learned the training set prior to damage. However, informal exploration of another model (the feed-forward auto-associator model of Tyler et al. 2000, of conceptual structure) has revealed that dependencies are possible. With that model, the behaviour of the network following the severing of connections was dependent on the distribution of weights prior to learning. (See Guest 2014, for a fuller exploration of how different training parameters affect the behaviour following damage of the Tyler et al. 2000, model.)

There are ways to resolve the question of which element (architecture or training set or training parameters or implementation of damage) is critical to explaining or accounting for a model’s behaviour. Most obviously one can explore the impact on a model architecture of different but related training sets, or explore whether different architectures trained with a common training set produce different behaviours. This kind of “sensitivity analysis” is essential to theoretical progress (cf. Cooper et al. 1996) yet rarely attempted. The only systematic work in this direction of which we are aware is that of Plaut and Shallice (1993a) with respect to acquired dyslexia and Bullinaria and Chater (1995) with respect to learning rules and exceptions.

### On Bridging Assumptions and the Implementation of Damage

Bridging assumptions, also known as linking propositions, have long been held to be necessary in order to relate levels of analysis within various subfields of cognitive neuroscience. (See, for example, Teller 1984, for an early analysis of different types of such assumptions within visual psychophysics.) Given such discussions, it might seem that any attempt to account for impairments due to neural damage within a cognitive model will necessarily require bridging assumptions that connect the cognitive and neural levels. In fact, the work reported here does not argue for the necessity of such bridging assumptions. The requirement depends upon whether the model is attempting to account for multiple distinct but related deficits (as in the case of the model of Lambon Ralph et al. 2007), and on whether model behaviour following damage is consistent across several forms of damage (as in the case of the model of Botvinick and Plaut^2004^).

In the case of Lambon Ralph et al. (2007), the bridging assumptions are (a) that connection severing results in the “dimming” of cognitive representations while weight perturbation through noise results in the “distortion” of those representations and (b) that the neural effects of semantic dementia can be modelling by connection severing while those of HSVE can be modelled by weight perturbation. Ueno et al. (2011) adopt similar assumptions, assuming without further comment that connection severing is an analogue of white matter damage while grey matter pathology can be modelled through the addition of noise to weights.

In the case of Botvinick and Plaut (2004), no specific bridging assumptions are required because several forms of damage (reflecting a range of potential bridging assumptions) yield qualitatively equivalent results, though the authors do not explicitly demonstrate this. In fact, it would be misleading if Botvinick and Plaut (2004) had provided bridging assumptions, because the insensitivity of their results to variation of the way in which damage is implemented means that the simulations reported therein could not distinguish between any of a range of potential assumptions that the authors might have made. This is also consistent with an analysis of the model purely at Marr’s “algorithmic and representational” level (Marr 1982).

One way to progress from Marr’s algorithmic and representational level to the implementational level would be to provide convergent evidence from multiple models across a range of domains for a specific implementation of damage. For example, and paralleling the arguments of the introduction, if models of a range of deficits, each attributable to (say) grey matter stroke in different neural regions, could be shown to reproduce the target behavioural profiles following damage implemented through the same process (e.g. perturbation of weights through the addition of noise), and if other implementations of damage only fit the behavioural profiles for a subset of models/deficits, then this would add weight to a bridging assumption relating the form of injury to the implementation of damage. However, the research surveyed in the introduction does not point to this. Instead, the range of approaches used to model successfully the behavioural effects of neural damage in different regions points to insulation of the algorithmic and representational level from the implementational level.

### Specification and Replication

Experimental psychology has recently received substantial criticism over a lack of replicability (e.g. Pashler and Wagenmakers 2012, and articles therein), with some highly cited results proving not to be robust. A range of reasons have been proposed for this lack of replicability, ranging from hypothesising after the fact to analysing data in multiple ways but reporting only those analyses producing significant results, and from failing to report full details of the experimental procedure or stimuli to downright fraud. Modelling is not immune from the underlying issues, as typically models are complex and not easily fully described within the confines of a standard article. The original description of the hub-and-spoke model of Rogers et al. (2004), for example, provides insufficient detail to allow replication (see Guest 2014), and this is more the rule than the exception.

Over the last decade, it has become common to make model code publicly available, and as a move towards open science, this should in our view be applauded. Indeed, without the materials of McClelland (2015), we would not have been able to conduct some of the studies reported here. However, making code available is not sufficient, in and of itself, to avoid a replication crisis within cognitive modelling. Most obviously, in addition to raw code one also needs values of all training parameter and the complete set of training items. But even this does not guarantee an understanding of what specific elements of a model (or indeed a training set) are critical to capturing the effects of interest. Equally, it does not prevent behaviour of an implementation being attributed to features that are in fact implementation details and not causally relevant to the generation or production of that behaviour.

Thus, as we have argued, rarely is it shown whether aspects of the training set or precise values of the training parameters (or indeed the way in which neurological damage is modelled, in the case of connectionist cognitive neuropsychology) affect a model’s behaviour. Whether this should be considered problematic arguably depends on how one views models and their function or purpose. If, on the one hand, a model’s purpose is essentially “proof of concept”—to demonstrate that a set of assumptions is consistent with some behaviour and hence a plausible candidate for the production of that behaviour, as in the case of the model of Botvinick and Plaut (2004)—then perhaps it is not problematic. If, on the other hand, a model’s purpose is more—to demonstrate that a set of assumptions *implies* the behaviour in question—then clearly it is.

Cooper and Guest (2014) argue on the above grounds for an implementation-independent approach to model specification, i.e. for the specification of a model’s critical assumptions without tying those assumptions to a specific implementation (also see Guest and Martin 2020). Such a specification requires a demonstration that implementation details in any particular instance do not determine the specification’s behaviour, and the complexity of psychological theory can obscure division between theory and implementation details (Cooper et al. 1996). Nevertheless this is important if we are to avoid over-extending theories beyond what can be supported by the empirical evidence.

There is a parallel here between direct replication and conceptual replication within the experimental literature (see, e.g. Stroebe and Strack 2014). Direct replication involves replicating precisely all aspects of an experiment (including stimuli, trial and block structure and experimental manipulation), whereas conceptual replication involves replicating an effect, typically with different stimuli and different experimental manipulations. Direct replications are important for establishing the existence of an effect, particularly given participants’ variability in responding and the resultant need for sophisticated statistical analyses, but conceptual replications are arguably more important for psychological theory because they imply that the factors leading to an effect are well understood, so much so that irrelevant factors may be abstracted away (and hence varied). Conceptual replication of computational studies is an important tool in ensuring that the causal processes behind effects attributed to a model are well understood. They are therefore of great theoretical importance.

### Modelling Aetiology and Aetiological Effects

There remains debate within the cognitive neuropsychological literature about whether aetiology (and hence type of damage), over and above lesion site, affects cognitive dysfunction. In the case of frontal dysfunction, Cipolotti et al. (2015) argue that lesion site and not aetiology is the determining factor. But cognitive deficits following frontal lesions are notoriously difficult to characterise in precise terms, so perhaps the reason why the argument can be made for frontal damage is simply because the subsequent impairments are ill-defined. The situation is even less clear cut in the case of deficits in the organisation of sequential action, where similar behavioural impairments can arise from neural damage with different origins and different loci (Schwartz et al. 1998b; Buxbaum et al. 1998). For the semantic dementia/HSVE distinction within semantic cognition, there is disagreement about the extent of lesion overlap (e.g. Noppeney et al. 2007; Lambon Ralph et al. 2017), and hence whether behavioural differences between the two are a consequence of lesion site (as might be the case if the overlap is only partial) or purely a function of type of damage (as might be the case if the overlap affects a critical area in different ways).

Relating these questions to connectionist cognitive modelling and the implementation of damage, it is too early to conclude that aetiological differences in patient populations cannot be captured through different approaches to modelling damage, but it can be concluded that attributing such behavioural differences to different forms of damage requires attention to factors beyond the basic organisation of the underlying model.

More specifically, there is no evidence that the behavioural consequences of semantic dementia might be most appropriately modelled within a connectionist network through weight disconnection or that the behavioural consequences of HSVE might be most appropriately modelled through weight perturbation. Equally, there is no support for the assumption of Ueno et al. (2011) that weight severing within a connectionist model reflects white matter (tract) lesions whereas weight perturbation reflects grey matter lesions. The assumption that specific computational implementations of damage reflect different types of neural damage is also not supported by the models surveyed in Table 1, where there is no consistency in the way behavioural impairments over a range of domains are accounted for within existing computational models.

This does not mean that different implementations of damage might not be appropriate for modelling different neurological impairments. Some deficits, even in the domain of semantic cognition, produce quite fine-grained behavioural impairments. Thus, patients described as semantic variant primary progressive aphasics, or svPPA, by van Scherpenberg et al. (2019), but in earlier literature classed as having an “access/refractory” deficit (see Warrington and Shallice 1979and Warrington and McCarthy 1983) tend to produce the same response (whether it be correct or erroneous) when presented with the same stimulus on successive occasions. This would seem to be a case where the modelling of damage in terms of weight perturbation (which would affect different trials in the same way) would be more appropriate than in terms of activation noise (which would affect different trials in different ways).^11^

### Attractor States and Representational Impairments

The existence of attractor states (and associated “basins of attraction”) within recurrent connectionist networks plays a central role in many explanations of normal and impaired behaviours. Category-sensitivity (i.e. different patterns of breakdown across animal and artefacts following simulated damage) is a key element of the recurrent hub-and-spoke model, and Lambon Ralph et al. (2007) explain the model’s behaviour following different forms of damage by appeal to differences between point attractors (i.e. attractors to which the network converges over time with fixed input) evoked by animal and artefact stimuli. Attractors (albeit path attractors, rather than point attractors) also play a critical role in the behaviour of the Botvinick and Plaut (2004) model of sequential action selection following damage, in that they support capture errors, whereby an action sequence appropriate for one task is captured by that appropriate for another task, or for a different point in the same task.

To account for differences between connection severing and weight perturbation, Lambon Ralph et al. (2007) call upon the mediating concepts of representational dimming and representational distortion. This is not the case in Botvinick and Plaut (2004), where similarity of representations within hidden unit space is the key explanatory concept. Despite the superficial appeal of representational dimming and representational distortion, our simulations found little evidence to support the concepts. Indeed, as noted earlier, reducing weights within the trained hub-and-spoke model by a constant proportion—a manipulation which would seem likely to produce representational dimming—does not produce the kind of erroneous behaviour attributed by Lambon Ralph et al. (2007) to representational dimming. The simulation evidence rather supports the view that multiple types of damage cause a more generalised representational impairment within both the hub-and-spoke model and the SRN model of sequential action (and by extension, within parallel distributed processing models more generally).

In fact, additional evidence suggests that such a representational impairment is independent of the concept of attractor. For example, an alternative feed-forward auto-associative model of semantic cognition, that of Tyler et al. (2000), shows category-sensitivity in the absence of recurrence (and hence in the absence of attractor states). This finding might appear to cast doubt on the role of attractors in the explanation of category-specific impairments offered by Lambon Ralph et al. (2007). Two points need to be borne in mind. First, Tyler et al. (2000) do not claim that their model shows the sensitivity to type of damage shown by the hub-and-spoke model (and simulations reported elsewhere attest to this: Guest, 2014). That is, mild damage of either type (severing of connections or adding noise to weights) yields qualitatively similar results in the Tyler et al. (2000) model but qualitatively different results in the hub-and-spoke model. Second, and as we have already discussed, the concepts of attractor dimming and distortion, following the severing of connections and the addition of noise to weights, respectively, do not fully capture the effects of the two forms of damage. Perhaps more critically, additional simulations reported in the Supplementary Materials, in which a feed-forward auto-associative network of the form used by Tyler et al. (but with 216 input/output units and 64 hidden units) was trained with hub-and-spoke patterns (both pattern set P1 and pattern set P2), showed sensitivity to the type of damage, with patterns corresponding to living things and artefacts behaving differently in response to severing of connections and the addition of noise to weights. As already noted, feed-forward auto-associative networks lack recurrent connections and hence any attractor structure. It is therefore unclear what, if any, explanatory role attractor states play in accounting for the sensitivity of the hub-and-spoke model to type of damage.

A further concept related to attractor space to which some authors have appealed is attractor density (e.g. Patterson and Hodges 1992; Plaut et al. 1996; Joanisse and Seidenberg 1999; Patterson et al. 2001; and Rogers et al. 2004). Thus, in the case of the hub-and-spoke model, Rogers et al. (2004) cite the density of attractor space (i.e. the number of distinct attractors within a given volume) as a factor mediating errors of omission, with omission errors being more common on naming tasks for items from sparse domains (artefacts) than items from dense domains (animals). This concept is largely orthogonal to that of dimming and distortion, because it does not concern the nature of the representational impairment resulting from different forms of damage. Moreover, it is not considered by Lambon Ralph et al. (2007) to be relevant to the primary difference in the semantic impairments of SD and HSVE patients (although Lambon Ralph et al. 2007, do imply that dimming and distortion yield similar effects in densely packed attractor spaces).

Our simulations question the concept of attractor density as being important in conditioning errors within the hub-and-spoke model, at least with respect to naming accuracy. Recall that for both P1 and P2 attractors for animal representations were more densely packed than attractors for artefact representations (at least as measured in terms of the mean Euclidean pairwise distance between attractors within each domain; see Table 5), but the effect of this was not consistent across damage type, with artefact knowledge being less robust than animal knowledge in the case of P2 and damage implemented through severing connections, but animal knowledge being less robust than artefact knowledge in the case of P1 with damage implemented through the perturbation of weights by the addition of noise.

### Beyond Generalised Damage within Distributed Learning Models

In this article, our specific focus has been on generalised damage within distributed connectionist models that learn from a set of exemplars, but the generic methodology of applying damage within an activation-based system, whether it be in the form of disconnection or scaling of weights or perturbation of weights or activations, has been successfully applied to other classes of model to simulate the cognitive effects of damage at the neural level. Equally, many simulation studies have considered effects of localised damage within topologically complex connectionist architectures. An important issue is therefore whether our concerns generalise to localised damage or to other classes of connectionist model.

Consider first localised damage within topologically complex network architectures (i.e. multi-layer networks with arbitrary connectivity between layers). Clearly, within such networks it is highly likely that damage, however implemented, to different sites will result in different behavioural impairments. Indeed, the existence of distinct impairments within a domain is frequently the motivation for more complex topologies. (See, for example, the so-called “triangle model” of Plaut et al. 1996, of reading and its impairments). However, our results suggests that it cannot be assumed that different implementations of damage at the same site within such a model will lead either to the same, or to distinct, behavioural impairments. The answer may depend in part on whether there exist regularities within subsets of the stimuli used to train the model, but ultimately the question can only be answered through computational experimentation.

Similar comments apply with respect to different classes of model. The class of interactive activation and competition (IAC) models, however, perhaps deserves special consideration given the number of models of this type. These models differ from the distributed learning models discussed above in several ways. Specifically, all units are typically semantically interpretable, connection weights between units are typically hard-wired or fixed, rather than learned, and processing typically involves activating a subset of units and then allowing the network to settle to a stable state where one unit from each set or layer is highly active, with the others in that set/layer being suppressed.^12^ There is therefore a sense in which the attractors of distributed network models correspond to the units of interactive activation and competition models. Studies to date suggest that different implementations of damage within such models can produce different behavioural outcomes (e.g. Cooper and Shallice 2000), though the differences may be subtle (Dell et al. 1997; Foygel and Dell 2000; Schwartz et al. 2006). The upshot of these considerations is that, as with other classes of model, it cannot be assumed that an IAC model’s behaviour, following lesioning, is independent of the implementation of lesioning.

### Cognitive Modelling and Cumulative Science

A final issue highlighted by the current work concerns the extent to which contemporary cognitive modelling research can be considered cumulative. Recall that a training set generated according to the template of Rogers et al. (2004) did not reproduce the critical results concerning naming deficits of semantic dementia patients of Lambon Ralph et al. (2007). This raises a critical question over whether the training set necessary (and arguably well-justified) for the latter work would reproduce all results of the original 2004 study. In other words, the Lambon Ralph et al. (2007) theory/results cannot be seen as a purely cumulative development of the theory/results of Rogers et al. (2004) because they are based on different training sets.

Unlike the hub-and-spoke model, the SRN model of sequential action selection of Botvinick and Plaut (2004) has not been the subject of additional simulation work. The question of accumulation of successful results is therefore not at issue. However, our results concerning the role of the training set in determining the errors to which the SRN model is prone (e.g. that omission errors are likely in impaired coffee making due to capture by tea making, which is similar to coffee making but does not involve cream-related actions), suggests that the critical simulation results of Botvinick and Plaut (2004) cannot be assumed to hold if the SRN model were extended or scaled to more tasks (and a larger training set).

More broadly, cumulative model development is a general issue that is not restricted to the modelling of neuropsychological deficits or connectionist modelling per se. It is particularly acute in the case of cognitive architectures, such as Soar (Newell 1990; Laird 2012) and ACT-R (Anderson 1983, 2007), where developments involving substantive changes to the underlying theory take place over decades and consequently where early results cannot be assumed to hold in later versions of an architecture.

Lack of accumulation should not necessarily be seen as undermining a research programme. Progress may well involve “unsolving” problems for which solutions had previously been found (Laudan 1978). It is therefore arguably a general problem for characterising progress within the philosophy of science. Perhaps what is most critical is clarity on the results that do and do not hold of any specific instance of a model within a broader research programme.

## Conclusion

When developing connectionist models of neuropsychological phenomena, neural damage may be simulated in a variety of ways. We have shown through two case studies that the way in which damage is simulated can, but does not necessarily, affect a model’s behaviour. Two general conclusions follow. First, it cannot generally be assumed that the way in which damage is simulated is merely an implementation detail. And second, it cannot be assumed from simulation of a specific form of damage (e.g. severing connections or perturbing weights through the addition of noise) that that form of damage is theoretically critical to the generation of the simulation results.

We can go further, however. The structure of the patterns on which a model is trained/tested is a critical factor in determining whether the model’s post-lesioned behaviour will be dependent on the form of damage. Thus, while we found limited support for concepts such as the dimming and distortion of attractors, despite their intuitive appeal, we found that a model’s response to different types of inputs following damage could nevertheless be sensitive both to correlational structure and relative magnitude in the model’s input domain, and to the specific form of damage. As a consequence, considerable care is needed when investigating how the behaviour of connectionist models breaks down if one is to reliably attribute findings to specific aspects of the model’s input/output domains or to a particular form of damage, and hence to be sure that findings are generalisable and characterised in such a way as to be fully replicable.

Replication within computational modelling is not merely an exercise in programming. It is, much more importantly, an evaluation of the specification as presented in the literature and of the modelling framework more generally. Connectionist neuropsychology, as with many scientific fields, must evaluate theoretical and computational proposals based on empirical findings and the properties of models. For this to occur, models must be described comprehensively yet in their simplest form.

Moreover, this work emphasises the importance of not ascribing to specific types of damage that which can be ascribed to any type of damage. Systematic analyses—such as examining networks before training (Guest and Love 2017), varying the type of damage (Plaut and Shallice 1993a) and conducting sensitivity tests (Cooper et al. 1996)—are needed before critical results can be appropriately ascribed to complex models. Without such “due diligence”, we cannot be sure where the credit for such results lies, or indeed if such results might hold for simpler models.

## Electronic supplementary material

(PDF 2.28 MB)

## Footnotes

1 Following the notation of Sartori and Lombardi (2004) and Sartori et al. (2007), we use corner brackets to denote concepts and angle brackets to denote features.

2 All three impairments are associated with damage to the temporal lobe, particularly on the left. However, the site of damage normally associated with the reverse dissociation is generally agreed to be more posterior than that involved in either SD or HSVE. Nevertheless, explaining all three deficits in a single model with different implementations of damage remains a theoretical possibility.

3 The precise pattern sets used in Rogers et al. (2004) and Lambon Ralph et al. (2007) were not available. The pattern set distributed by McClelland (2015) is derived from that used in the simulations reported in Rogers et al. (2004; T. T. Rogers, personal communication, August, 21, 2012), though our results suggest it is not identical to that used in the earlier work.

4 Given that this algorithm can be implemented in multiple ways, the simulations reported here use the implementation (i.e. the precise codebase) of the model distributed with the PDPTool software (for implementational and algorithmic details see McClelland^2015^).

5 In order to ensure that our results from P2 were not a statistical aberration, the procedure was repeated with twenty different versions of P2, each generated according to the probabilistic template of Rogers et al. (2004). Consistent behaviour was found, and unless otherwise stated the results presented in this section relate to one representative instance of P2.

6 Rogers et al. (2004) decompose the 112 verbal features into 64 perceptual, 32 functional and 16 encyclopaedic features. However, their template provides details of only 61 perceptual features but 18 encyclopaedic features. Given that the precise training set used in Rogers et al. (2004) was unavailable (see footnote 3), we extrapolated three further perceptual features by duplicating the rightmost three features of the template, while ignoring the two rightmost encyclopaedic features. These adjustments do not appear to affect the similarity structure of categories or domains (see Fig. 2) and further simulations suggest that the results reported here hold over reasonable alternative approaches. (See also the Supplementary Materials for additional investigations of the consequences of training with different pattern sets.)

7 Note that the number of features present for fruits varies between P1 and P2. Fruits are argued to pattern differently in the behavioural data from animals and artefacts, and are neither considered in any of the simulations reported in this paper nor in the equivalent ones in Rogers et al. (2004).

8 PDPTool provides a discrete approximation to a continuous-time conceptualisation of backpropagation through time. Within that approximation, each processing cycle is divided into a number of ticks (and so the more ticks per cycle the better the approximation), and activation at each tick is a weighted sum of activation due to recurrent excitation and activation on the previous tick. The PDPTool implementation of the hub-and-spoke model uses 4 ticks per cycle. The 7 processing cycles described in the main text therefore correspond to 28 ticks.

9 There is a separate debate about the role of left temporoparietal cortex and subcortical structures—specifically the basal ganglia—in the control of sequential behaviour. See, for example, De Renzi and Lucchelli (1988), and Rumiati et al. (2001) versus Buxbaum et al. (1998), Schwartz et al. (1998a), and Schwartz et al. (1998b). We consider damage to these structures to give rise to a related but distinct disorder of action selection. As such, we see this as a separate issue that bears upon the adequacy of the SRN model as a model of action control more generally but not as a model of ADS.

10 This observation parallels the systematic analysis of the behaviour of different groups of patients with frontal damage—stroke patients, low/high grade tumour patients, and meningioma patients—by Cipolotti et al. (2015), which demonstrated independence of aetiology in the effects of frontal damage on common “frontal” tests.

11 See Howard et al. (1984), for a detailed analyses of aphasic response consistency, and Gotts and Plaut (2002), for a model of access/refractory deficits.

12 IAC models support, in addition to all of the forms of damage considered earlier in this paper, a further form of damage—activation decay.
