# Supplementary material for: On Simulating Neural Damage in Connectionist Networks
Source: Comput Brain Behav. 2020 Jun 30;3(3):289–321. doi: 10.1007/s42113-020-00081-z (PMC7381482; doi:10.1007/s42113-020-00081-z)
Supplement: Supplementary file 1 — (PDF 2.28 MB) [file 42113_2020_81_MOESM1_ESM.pdf]

On Simulating Neural Damage in Connectionist Networks: Supplementary Materials

Olivia Guest
Research Centre on Interactive Media, Smart Systems and Emerging Technologies — RISE, Nicosia, Cyprus
& Department of Experimental Psychology, University College London, UK

Andrea Caso
Department of Psychological Sciences, Birkbeck, University of London, UK

Richard P. Cooper
Department of Psychological Sciences, Birkbeck, University of London, UK

The Hub-and-Spoke Model of Rogers et al. (2004)

In addition to the simulations reported in the main text, and in order to better understand the factors that affect relative preservation of domain knowledge, we investigated the behaviour of the hub-and-spoke model following damage when trained with several additional pattern sets. We refer to these as P5, P6, P7 and P8. (Pattern sets P3 and P4 were based on the same template as P2, and are not reported here.) The pattern correlation matrices for P5, P6, P7 and P8 are shown in Figure 1, while the norms for each category of each pattern set are given in Table 1. These matrices and figures confirm that the pattern sets possess the intended correlational and norm structure as described below.

P5 is designed to explore how differences in vector norms across categories affect the network's behaviour. The patterns are generated from the standard template, but then non-name features are each scrambled, so animal and bird vectors still contain more "1"s than the other categories, but there is no longer any correlational structure between any of the vectors. (Note the lack of correlation structure in Figure 1A, while the vector norms for P2 and P5 given in Table 1 are similar for each of the categories.)

In P6, all categories/domains have similar / indistinguishable norms, but one domain (animals) has no correlation structure while the other (artefacts) has the structure given by the Rogers et al. (2004) template. (To see this, note the correlation structure in Figure 1B, while the vector norms for P2 and P6 given in Table 1 are similar for each of the categories.)

P7 is based on the original template (like P2), plus a few more shared features for birds and mammals so that their correlations are more like those in P1. The hypothesis is that P7 will replicate P1 because the structure in animals will result in a denser representation of animals that will consequently be less robust to damage. The hypothesis is that this should shift the animal lines in both the weight perturbation and connection severing graphs to the left from where they were for P2, but without moving the artefact lines, making the graphs look more like those for P1.

As it happens P7 yields results that are quite similar to P2. The problem appears to be that in P7 animal names have even larger norms than in P2 (see Table 1), so the difference in norms between animals and artefacts is even greater than in P1 or P2, and this works against the effect. P8 therefore has norms like P1 but stronger correlations within animals. It is a relatively minor modification of the Rogers et al. (2004) template that is designed to behave as P1.

Simulation results for the two forms of damage considered by Lambon Ralph, Lowe, and Rogers (2007) are shown in Figure 2 and Figure 3. Figure 2 (left) shows that domain differences in the size of vectors makes a difference to how the network treats the exemplars, even in the absence of within-category correlations. Animals, which have larger norms, are better preserved following connection severing than artefacts, while weight perturbation affects both domains similarly. Figure 2 (right) shows that corelational structure within artefacts (in the absence of similar structure for animals) results in artefact knowledge being more prone to lose than animal knowledge, regardless of damage type. These simulations strongly suggest that it is not the correlational structure that is differently sensitive to type of damage, since that differentiation happens even without correlational structure. Rather, it is differences in the norms of the vectors. Correlational structure merely adds to the effect (roughly equally in both domains), that is present when no correlational structure exists.

Figure 3 (left) shows that, as discussed above, the manipulation used to generate P7 was only partially successful. Like P2, this manipulation results in an animal advantage when connections are severed and an artefact advantage when weights are perturbed. Note though that in P7 animal names have even larger norms than in P2 (see Table 1), so the difference in norms between animals and artefacts is even greater than in P1 or P2, and this works against the effect. Consistent with this, Figure 3 (right) shows the results obtained from pattern set P8. These replicate those of P1, and provide further support for the proposal that domain-differences in norms, in addition to differences in within-

Category	P1	P2	P5	P6	P7	P8
Birds	6.498	5.985	6.124	5.835	6.771	6.341
Mammals	6.422	6.059	6.099	5.504	6.582	6.363
Fruits	6.894	5.585	4.563	5.235	5.560	5.262
Tools	5.023	4.913	4.974	5.063	5.061	5.102
Vehicles	5.234	5.346	5.418	5.361	5.417	5.411
HH Objects	4.741	4.960	4.978	4.905	4.613	4.777

Table 1
Vector norms for each category for each of the pattern sets explored in this section. Norms for P1 and P2 are given for comparison.

category correlational structure, play a critical part in producing the effect reported by Lambon Ralph et al. (2007).

The Conceptual Structure Model of Tyler et al. (2000)

In this section we compare the effects of two different types of damage (lesioning connection weights and adding noise to connection weights) in three implementations of a second model of semantic cognition, the conceptual structure model of Tyler, Moss, Durrant-Peatfield, and Levy (2000).

One implementation is our best estimate at the actual implementation of Tyler et al. (2000), in that it uses precisely the same architecture (a feedforward autoassociator with 24 input and output units and 20 hidden units) and learning algorithm (standard backpropagation). It also uses the same set of patterns for training/testing as described by Tyler et al. (2000) and the same learning rate (0.25). Tyler et al. (2000) do not specify all aspects of their implementation, however, and our implementation may have differed in four ways:

1. Tyler et al. (2000) do not specify the initial distribution of network weights. The simulations reported here assume that weights are initialised to small random values uniformly sampled from the range [−0.01, +0.01]. Investigation of network behaviour revealed that this apparently insignificant detail matters. If the initial weight range is smaller then the cross-over in Figure 4 below moves to the left. If it is bigger, it moves to the right, and when the range is too large (e.g., [−0.10, +0.10]) the cross-over does not occur at all.

2. Tyler et al. (2000) do not state the precise condition when training is terminated, stating only that "Training was stopped after a mean of 984 presentations of the complete target set when the summed squared error for each vector was about .01." (p. 214). The simulations reported below assume that training is terminated after 1000 epochs.

3. Tyler et al. (2000) do not state whether weights were updated by item or by epoch. The simulations reported below assume the latter, though this appears to make little difference to the results.

4. Lastly, Tyler et al. (2000) report using a momentum value of 0.90. Using backpropagation as specified by Hertz, Krogh, and Palmer (1991, pp. 116, 117, 123), we found that the network was unable to learn the full pattern set with such a high value, but different implementations of backpropagation characterise momentum slightly differently (with some, for example, presenting essentially the same parameter as a difference from 1.00, and others using it, or the difference, to scale the contribution to weight changes from the current epoch). We found that a value of 0.10 (i.e., 1.00−0.90) allowed us to reproduce the general patterns reported by Tyler et al. (2000), though the network was able to learn the pattern set adequately with values in the range 0.00 to about 0.65.

The second implementation explored the role of attractors by adding recurrent (i.e., hidden to hidden) connections to the basic autoassociator architecture. There were again 24 input and output units, and 20 hidden units, with full connectivity between units in the hidden layer. The network was trained using epochwise backpropagation through time (Williams & Zipser, 1995, eqs. 17–20) on each pattern for 10 cycles. Throughout training inputs were clamped to the appropriate input pattern for the full 10 cycles. The learning rate was set to 0.025 (i.e., the learning rate of the feedforward network divided by the number of cycles per pattern). Beyond this, the network setup and testing was identical to that of the feedforward network. We refer to this implementation as the clamped Recurrent Autoassociative Network (RAN_C) implementation.

The final implementation, which we refer to as the unclamped Recurrent Autoassociate Network (RAN_U) implementation, was again a recurrent network trained as an autoassociator with epochwise backpropagation through time, but in this version of the model the input was clamped for four cycles and then set to zero. In training, the network then continued processing for a further six cycles, with output clamped for the full 10 cycles to the desired output. In testing, the network was allowed to cycle until settled. The learning rate was again set to 0.025, but training was extended to a total of 5000 epochs. In order to learn the task

this final network needed to learn attractor states that could be maintained in the absence of input. This form of network was considered in order to argue against any suggestion that the attractors of the clamped RAN were driven only by the fixed input and were not true attractors.

A series of simulation studies were run in which the three models were trained and damaged either through lesioning a proportion of connections (as in the original work) or through the addition of normally distributed random noise to the connection weights (i.e., weight perturbation). In each case 21 levels of damage were considered, with the proportion of weights lesioned ranging form 0% to 100% in increments of 5% and the standard deviation of noise ranging from 0.000 to 3.000 in increments of 0.015. The maximum level of noise was chosen to approximate the error rate resulting from a 100% lesion to weights. For comparison, the above training procedures led to networks with weights in the approximate range [−5, +5], so the maximum standard deviation of noise was approximately 60% of the maximum absolute value of the weights.

As shown in Figure 4, lesioning and noise affect distinctive features in the two domains in different ways, but only at very high levels of damage.[1] For low to moderate damage, both lesioning and noise affect distinctive features of artefacts more than distinctive features of animals. When damage is severe, however, it appears that this effect reverses when damage is implemented by lesioning connections. The reversal does not occur, however, when damage is implemented by adding noise to connections. Arguably this effect is unreliable, however, as the point of reversal depends on the initial network weights, with the point at which the cross-over occurs in the lesioned model moving to the right as the standard deviation of the initial weights increases.

Figure 5 shows that type of damage makes no qualitative difference to shared and distinctive features of animals. In both cases, mild-to-moderate damage harms distinctive features more than shared features, while the effect reverses for more severe damage. This holds true for both forms of damage.

For both forms of damage the effect of damage on shared features is more severe for artefacts than animals, though the size of the effect is clearly greater for lesioning of weights than for the addition of noise to weights (cf. Figure 6).

Figure 7 contrasts the effects of different forms of damage on the functional features of animals and of artefacts. In both cases, the features are more robust in animals, though at high levels of noise (but not high levels of lesioning) the difference disappears.

Figure 8 shows identification accuracy for animals versus artefacts, with both forms of damage. For lesioning, it appears that artefact identity is relatively well-preserved in comparison to animal identity for mild-to-moderate damage, but the pattern reverses for severe lesioning. This reversal is not apparent in the case of noise, where high levels of noise results in similar levels of identification failure across domains.

Finally, Figure 9 shows the breakdown of errors in each domain. The 16 patterns of Tyler et al. (2000) are designed so as to correspond to eight objects within two domains (living things and artefacts) and the eight objects in each domain are intended to correspond to four objects within two categories (e.g., mammals and birds in the living things domain, vehicles and tools in the artefacts domain). Given this composition of the training set, errors may be classified as within-category errors (e.g, calling a robin an eagle), between-category errors (calling a robin a dog), or cross-domain errors (calling a robin a truck). The figure shows just the within-category and between-category errors.

Tyler et al. (2000) argue that two characteristics of their corresponding graph (their Figure 8) are critical. First, "The network consistently makes more within-category errors for living things than for artefacts" and "In contrast, between-category errors for artefacts occur with mild levels of lesioning, earlier than the first between-category errors for living things." (Tyler et al., 2000, p. 222–223). While there are clear differences between the left panels (connection severing) and right panels (weight perturbation) in Figure 9, all graphs show this basic pattern. In particular, at lower levels of damage, it is living things that are most prone to errors (consistent with Figure 8), and those errors are within-category errors (e.g., a bird is incorrectly identified as being some other bird). As the level of damage increases (regardless of type), errors also occur with artefacts, though again these errors are (with lower levels of damage) within-category errors. As the level of damage increases, both forms of damage lead to between-category errors (e.g., incorrectly identifying a bird as a mammal and incorrectly identifying a vehicle as a tool). The relative frequencies of the types of errors for each domain is largely preserved (i.e., for mild to moderate damage, within-category errors exceed between-category errors, and this is true for both domains and both types of damage). Differential effects of the different forms of damage are only seen with extreme damage, where extreme lesioning results in a tendency to produce a single response (which is always a living thing, meaning that all artefact errors are between-domain errors and four out of eight living things responses are between-category errors, with three out of eight being within-category errors and the remaining response being correct).[2] In contrast, extreme

[1]As in Tyler et al. (2000), bias weights were not affected by damage.

[2]This behaviour — a preference for a living things response — can be traced back to the fact that the bias units are not lesioned. Consequently 100% connection severing maintains the bias connections, which means that the output for any input reflects the baserate of each output feature. This favours living things over artefacts.

weight perturbation results in substantial (between-subject) variability of responding, such that either response (living thing or artefact) may be given for either type of stimulus.

To summarise these simulations, direct comparison of lesioning connections and adding noise to weights in the conceptual structure model does show some differences between the effects of the two forms of damage, but those difference only occur at extreme levels of damage (e.g., greater than 75% connection severing compared to weight perturbation with standard deviation of greater than 2.0). This is not surprising when one considers that extreme levels of connection severing will result in the network's response being independent of its input. This will not be the case with extreme levels of weight perturbation. At the same time, reviewing the figures, it is apparent that *there are no substantive differences between the architectures (feedforward or recurrent) following breakdown*. Both feedforward and recurrent architectures respond to lesioning in the same way (and regardless of whether inputs are clamped or not during settling), and the same is true for the response of the different architectures to noise. It follows that any difference in network behaviour following damage due to connection severing versus weight perturbation cannot be attributed to attractor structure, as the feedforward network does not possess attractors.

A Hybrid Model of Semantic Cognition

In this section we consider the behaviour of a model with the architecture of the conceptual structure model (i.e., a feedforward autoassociate network), but the training set of the hub-and-spoke model (or more precisely, pattern sets P1 and P2 as used in the main text). This hybrid model is of interest as it allows us to tease apart the structural properties of the hub-and-spoke training sets from the recurrent aspects of the hub-and-spoke model.

Following the approach of Tyler et al. (2000) and given that the "hub" of the hub-and-spoke model consists of 64 units, the hybrid architecture considered here consists of 216 input units and 216 output units (i.e., one unit per feature of the hub-and-spoke pattern sets) and 64 hidden units. In order to auto-associate the inputs with the outputs, the model is therefore required to compress the representations of 216-bit items in the training set to 64 bits. This is assumed to be feasible given that it is the task successfully performed by the hub-and-spoke model. With this architecture, the hybrid model was trained using standard back-propagation with the same parameter values used for training the Tyler et al. (2000) model (i.e., initial weight range of [−0.01, +0.01], learning rate of 0.25, training for 1000 epochs, weight update by epoch, and momentum of 0.10).

Three hundred instances of the model were trained with pattern set P1 and a further three hundred were trained with pattern set P2. Each instance was then damaged as in the earlier studies, i.e., through the severing of a proportion of connections or the perturbation of weights through the addition of random noise. Figure 10 shows identification accuracy for living things and artefacts while Figure 11 shows naming accuracy for animals and artefacts as a function of level of damage, for each type of damage and each training set (cf. Figure 8, Figure 2, and Figure 3). Figure 10 uses the identity criterion of Tyler et al. (2000), i.e., nearest trained pattern to the output (by Euclidean distance), while Figure 11 uses the naming criterion of Lambon Ralph et al. (2007), i.e., most active name unit in the output.

What is immediately apparent from the figures is that the type of damage affects the networks' behaviour following damage, and while artefact identification is arguably slightly more robust for P2 than for P1, this difference is very small compared to the effect of type of damage. Critically, when damage is implemented through the addition of noise to weights, identification of living things is more impaired than identification of artefacts, but when damage is implemented through the severing of connections the dissociation is reversed, with identification of living things being better preserved. While none of the previous simulations shows such robust living things knowledge, the patterns of impairment show that attractors per-se are not the critical feature of the hub-and-spoke model that results in sensitivity to type of damage, as that sensitivity is present in a model without attractors. The key feature appears to be the structure in the training patterns and the "representational compression" achieved within the auto-associative network.

The Recurrent Network Model of the Control of Routine Sequential Action of Botvinick and Plaut (2004)

A further series of simulation studies was conducted in order to investigate the effects of different types of damage on the simple recurrent network of Botvinick and Plaut (2004). As a starting point we used the reimplementation of Botvinick and Plaut (2004) reported in Cooper and Shallice (2006). Botvinick and Plaut (2004) implemented damage by adding normally distributed random noise to context units. In the simulations reported here we consider this and two other forms of damage: adding normally distributed random noise to weights, and lesioning a proportion of weights. As discussed in the main text, several simulations were run with the aim of reproducing the key figures in Botvinick and Plaut (2004).

Figure 12 shows survival plots for the various simulations when the networks are trained, damaged and then instructed to perform each of the tasks on which it was trained. In all cases errors tend to occur primarily at subtask boundaries, as evidence by the sharp performance drops for the coffee preparation task at steps 10/11, 21/22 and 32/33, and similar performance drops for the tea preparation task at steps 10/11

and 15/16.[3] Figure 13 shows a breakdown of errors by type as a function of level of damage. In all cases, low levels of damage result primarily in subtask errors, while high levels of damage result in within-subtask errors. Figure 14 shows how damage affects the type of errors (in terms of independent actions, crux action errors and non-crux action errors) made by the model. In all cases except for weight scaling, independent errors are most common while crux errors are least common. This holds over both tasks and six of the seven implementations of damage. Figure 15 shows the number of errors per trial at various levels of damage, for each form of damage. In all cases, low levels of damage result in a range of errors (including different types of errors of commission), while high levels of damage result in increasing numbers of omission errors. In all cases there are differences between the precise patterning of errors when preparing coffee versus preparing tea, but those differences are not qualitatively different for the different implementations of damage. Thus, and to summarise, the simulations of different forms of damage within the Botvinick and Plaut (2004) SRN support our hypothesis: with the exception of weight scaling, the various different forms of damage are qualitatively equivalent in terms of their effects on the network's behaviour.

References

Botvinick, M. M., & Plaut, D. C. (2004). Doing without schema hierarchies: a recurrent connectionist approach to normal and impaired routine sequential action. *Psychological Review*, *111*(2), 395-429.

Cooper, R. P., & Shallice, T. (2006). Hierarchical schemas and goals in the control of sequential behavior. *Psychological Review*, *113*(4), 887-916.

Hertz, J., Krogh, A., & Palmer, R. G. (1991). *Introduction to the theory of neural computation*. Reading, MA: Addison-Wesley.

Lambon Ralph, M. A., Lowe, C., & Rogers, T. T. (2007). Neural basis of category-specific semantic deficits for living things: evidence from semantic dementia, HSVE and a neural network model. *Brain*, *130*(4), 1127–1137.

Rogers, T. T., Garrard, P., McClelland, J. L., M.A. Lambon Ralph, Bozeat, S., Hodges, J., & Patterson, K. (2004). Structure and deterioration of semantic memory: A neuropsychological and computational investigation. *Psychological Review*, *111*(1), 205–235.

Tyler, L. K., Moss, H. E., Durrant-Peatfield, M. R., & Levy, J. P. (2000). Conceptual structure and the structure of concepts: A distributed account of category-specific deficits. *Brain and Language*, *75*(2), 195–231.

Williams, R. J., & Zipser, D. (1995). Gradient-based learning algorithms for recurrent networks and their computational complexity. In Y. Chauvin & D. E. Rumelhart (Eds.), *Back-propagation: Theory, architectures and applications* (pp. 433–486).

[3]The pedantic reader will note that the results of our exact replication here and in later figures report the standard deviation of activation noise rather than the variance, as reported by Botvinick and Plaut (2004). Quantitative aspects of our reimplementation suggest that Botvinick and Plaut (2004) conflated variance with standard deviation.

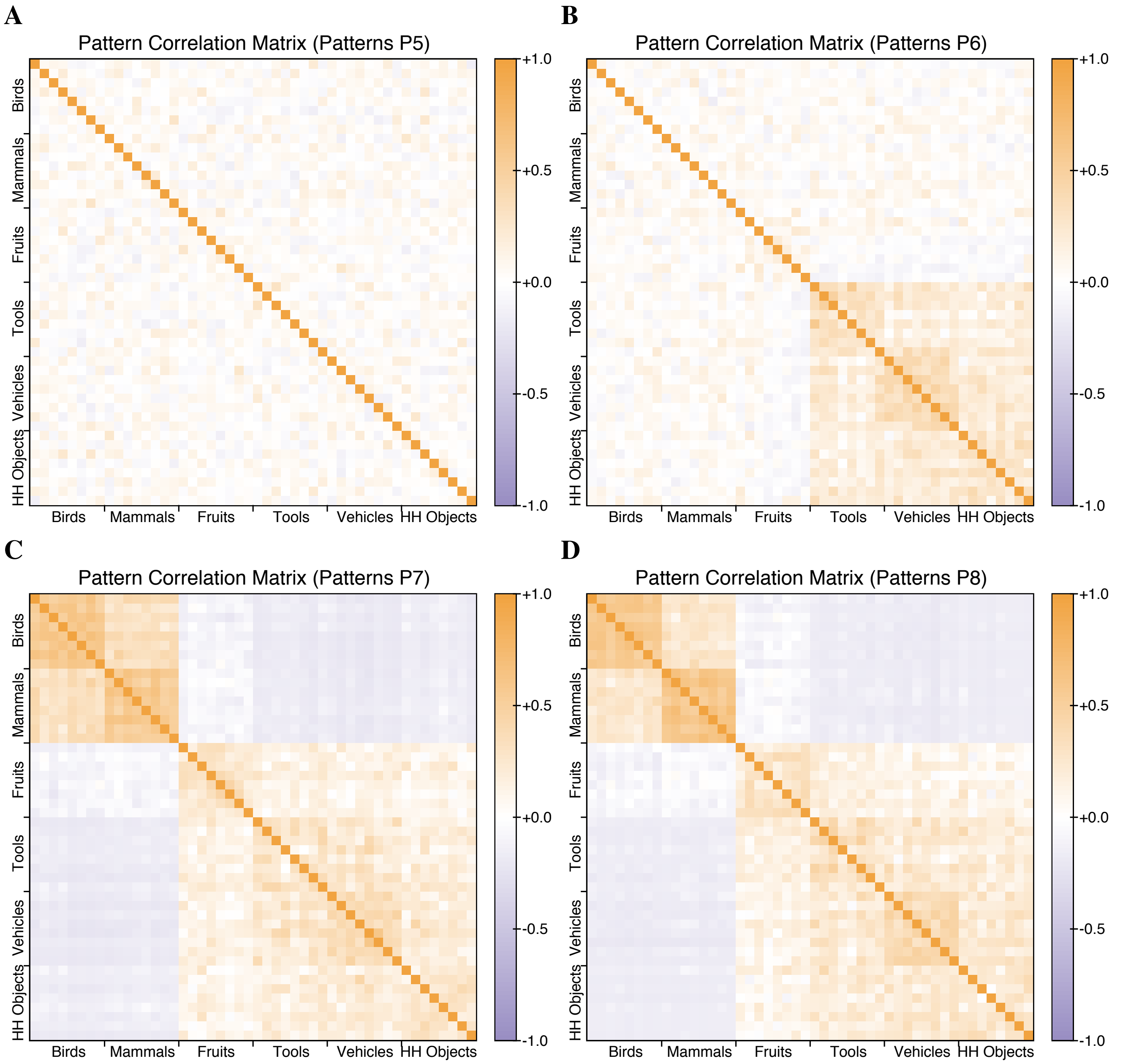

Figure 1. Correlation matrices showing the similarity space of the four additional training sets. The similarity space of **A)** pattern set P5; **B)** pattern set P6; **C)** pattern set P7; and **D)** pattern set P8; Pattern set P5 features no within-category or between-category correlations, while pattern set P6 has within-category correlation only within the artefact categories. Pattern sets P7 and P8 have stronger within-category correlations within animal domains than artefact domains.

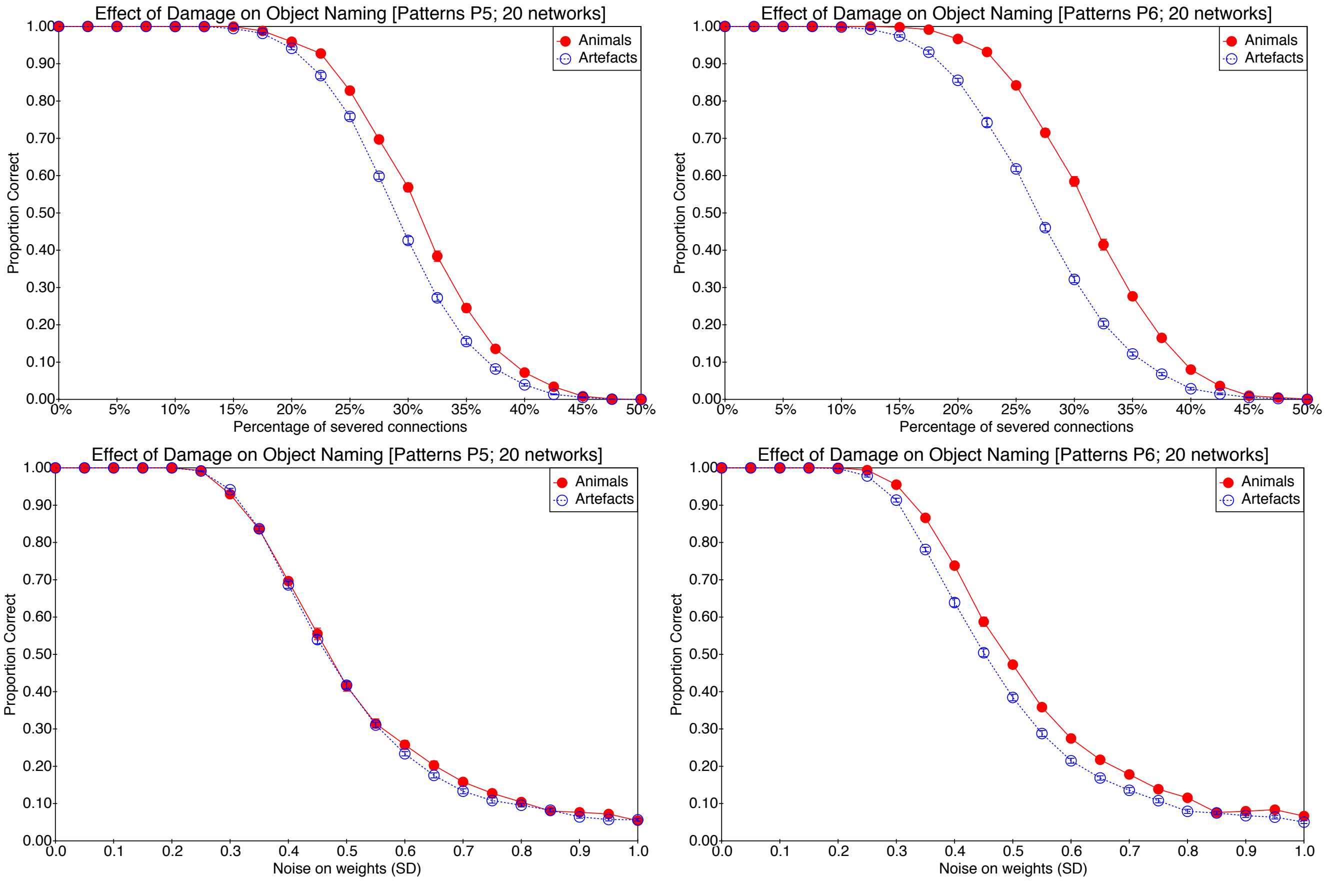

Figure 2. The effect of damage (severing connections or perturbing weights) on naming accuracy for animals and artefacts when the hub-and-spoke model is trained with P5 (left) and P6 (right). Each data point represents mean performance on 10 trials for 20 virtual subjects. Error bars correspond to ±1 SE.

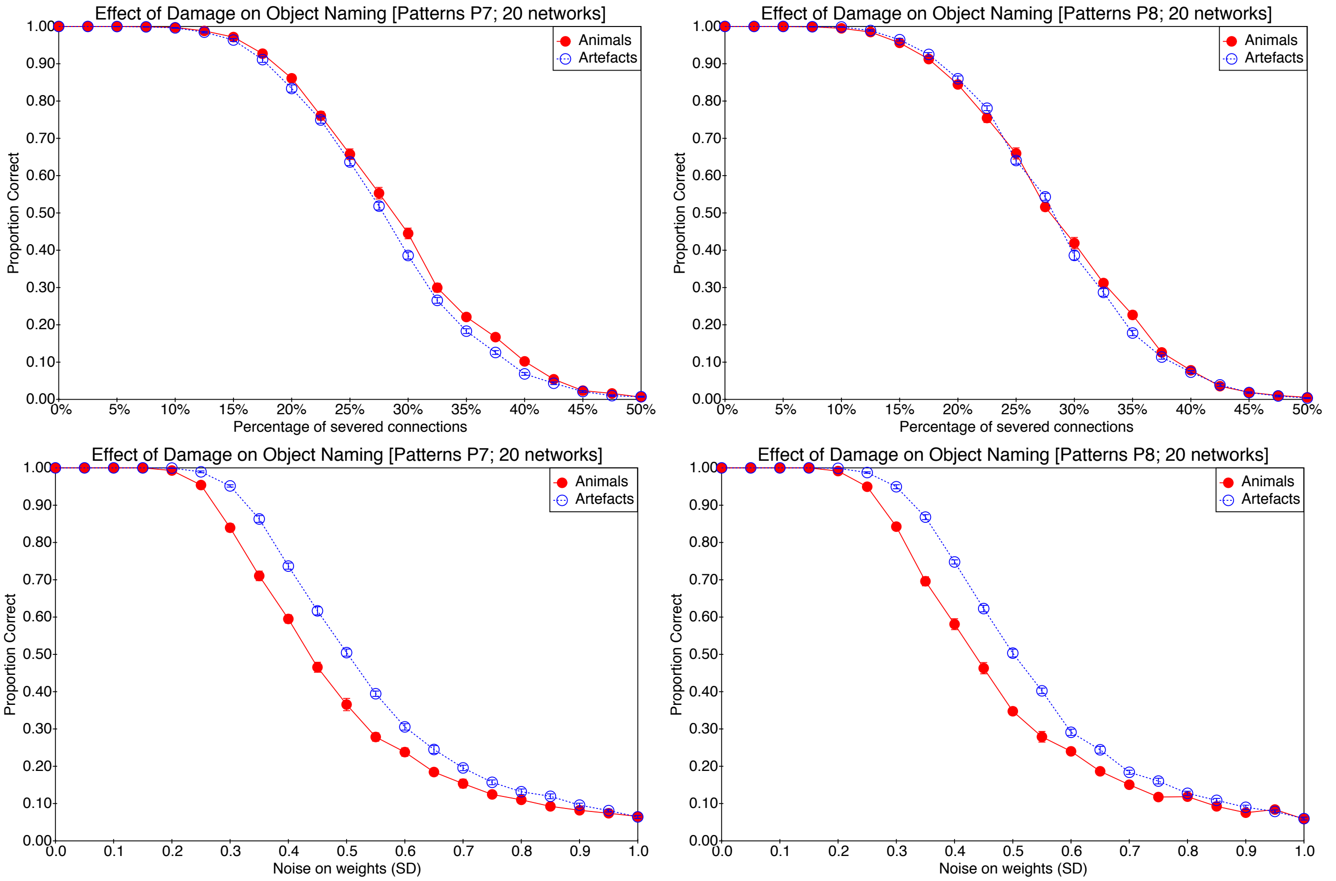

Figure 3. The effect of damage (severing connections or perturbing weights) on naming accuracy for animals and artefacts when the hub-and-spoke model is trained with P7 (left) and P8 (right). Each data point represents mean performance on 10 trials for 20 virtual subjects. Error bars correspond to ±1 SE.

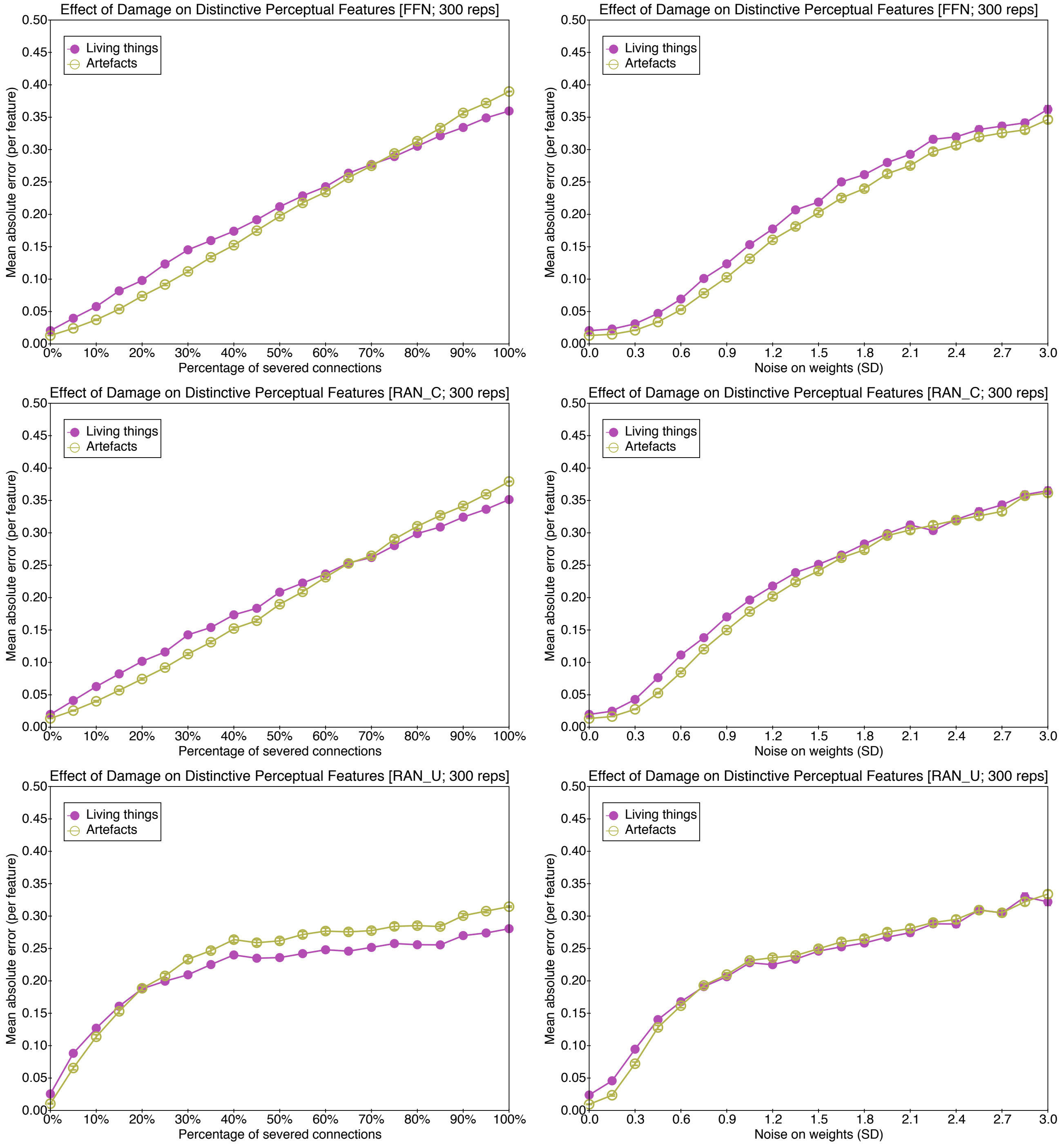

Figure 4. Effect of damage on distinctive features in animals versus artefacts in the feedforward network (FFN; upper), clamped recurrent attractor network (RAN_C; centre) and unclamped recurrent attractor network (RAN_U; lower) implementations of the conceptual structure model of Tyler et al. (2000). Left: Effect of lesioning connections. Right: Effect of adding noise to connection weights.

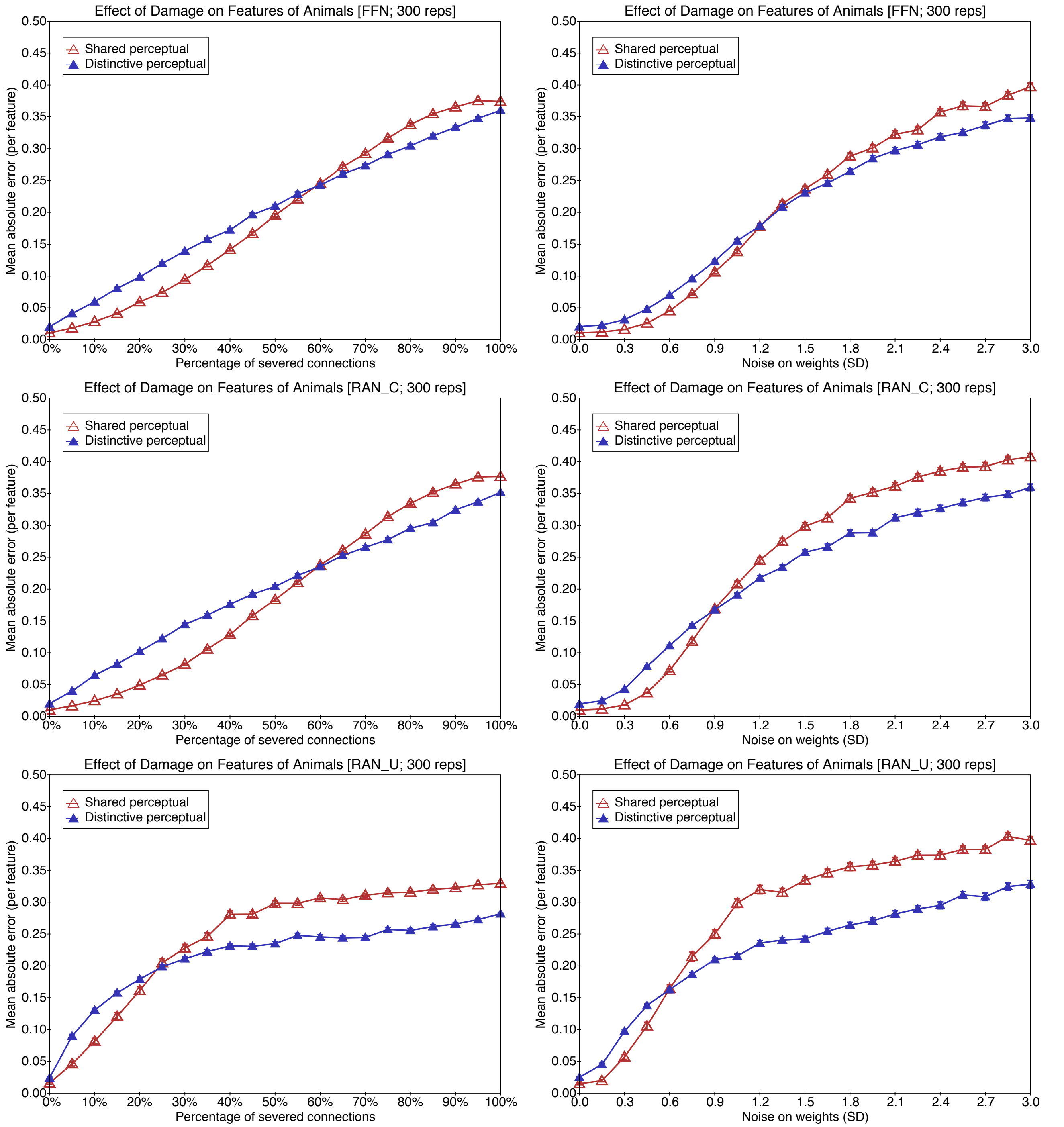

Figure 5. Effect of damage on shared versus distinctive perceptual features of animals in the feedforward network (FFN; upper), clamped recurrent attractor network (RAN_C; centre) and unclamped recurrent attractor network (RAN_U; lower) implementations of the conceptual structure model of Tyler et al. (2000). Left: Effect of lesioning connections. Right: Effect of adding noise to connection weights.

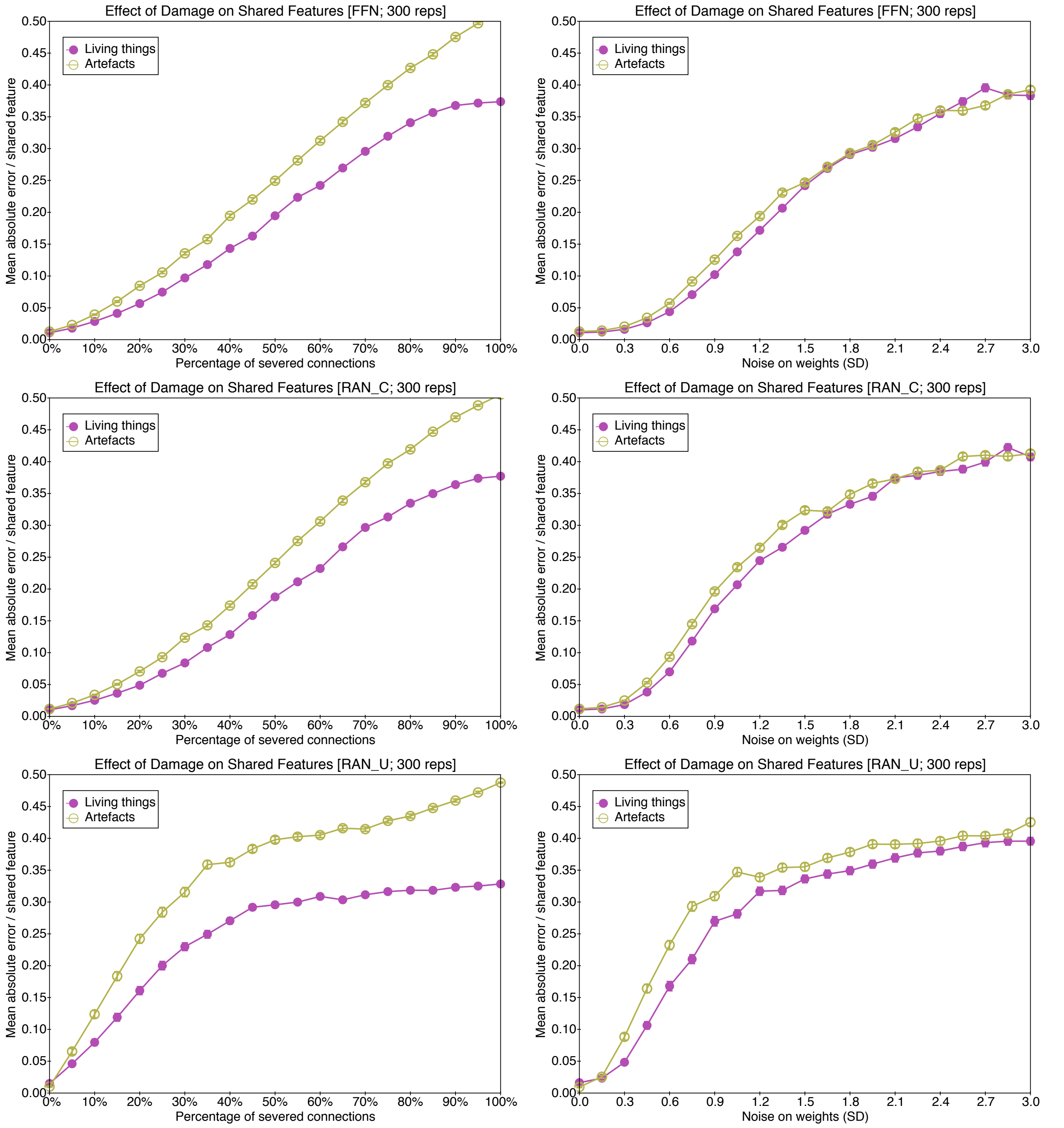

Figure 6. Effect of damage on shared features of animals and artefacts in the feedforward network (FFN; upper), clamped recurrent attractor network (RAN_C; centre) and unclamped recurrent attractor network (RAN_U; lower) implementations of the conceptual structure model of Tyler et al. (2000). Left: Effect of lesioning connections. Right: Effect of adding noise to connection weights.

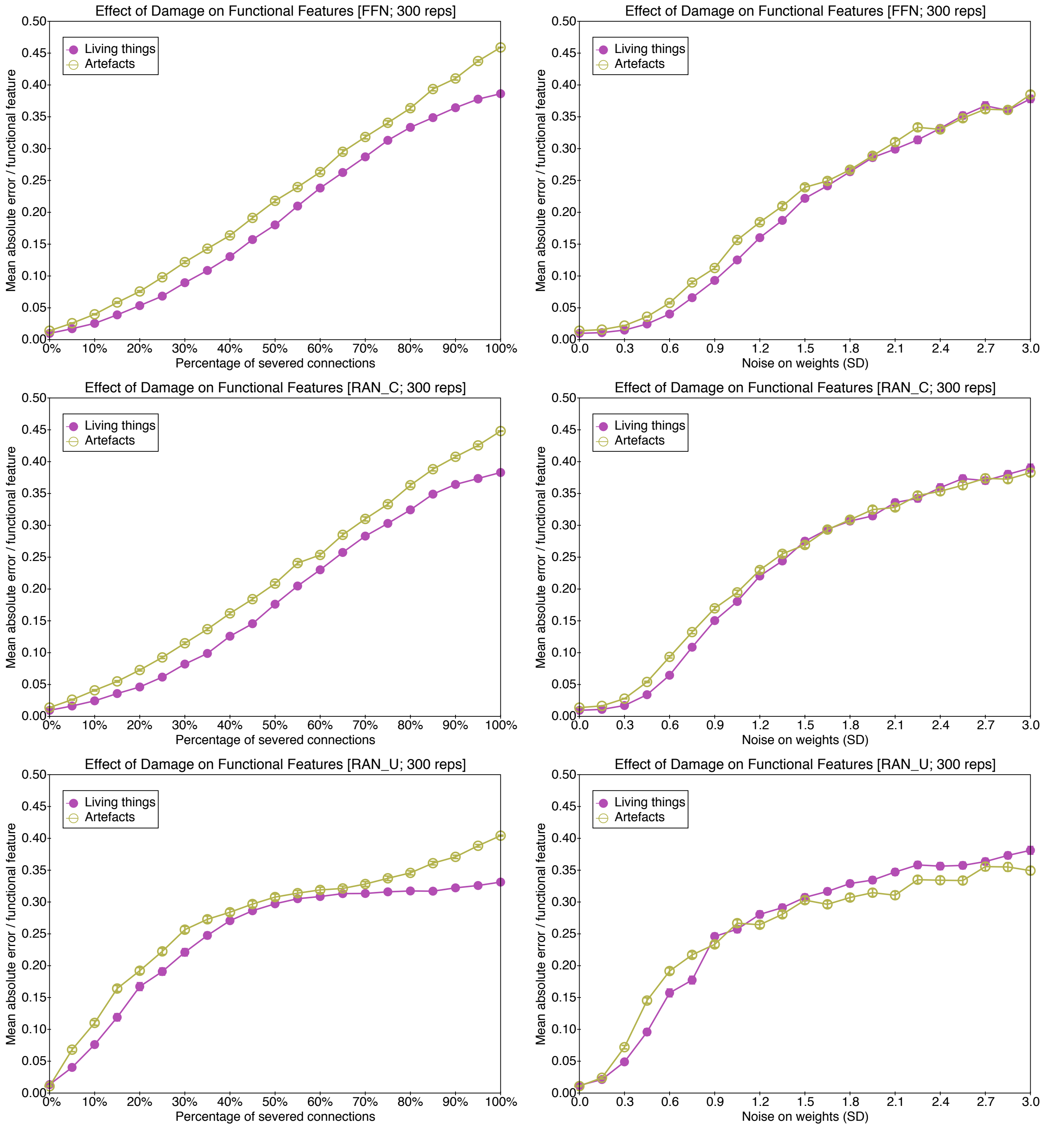

Figure 7. Effect of damage on functional features of animals and artefacts in the feedforward network (FFN; upper), clamped recurrent attractor network (RAN_C; centre) and unclamped recurrent attractor network (RAN_U; lower) implementations of the conceptual structure model of Tyler et al. (2000). Left: Effect of lesioning connections. Right: Effect of adding noise to connection weights.

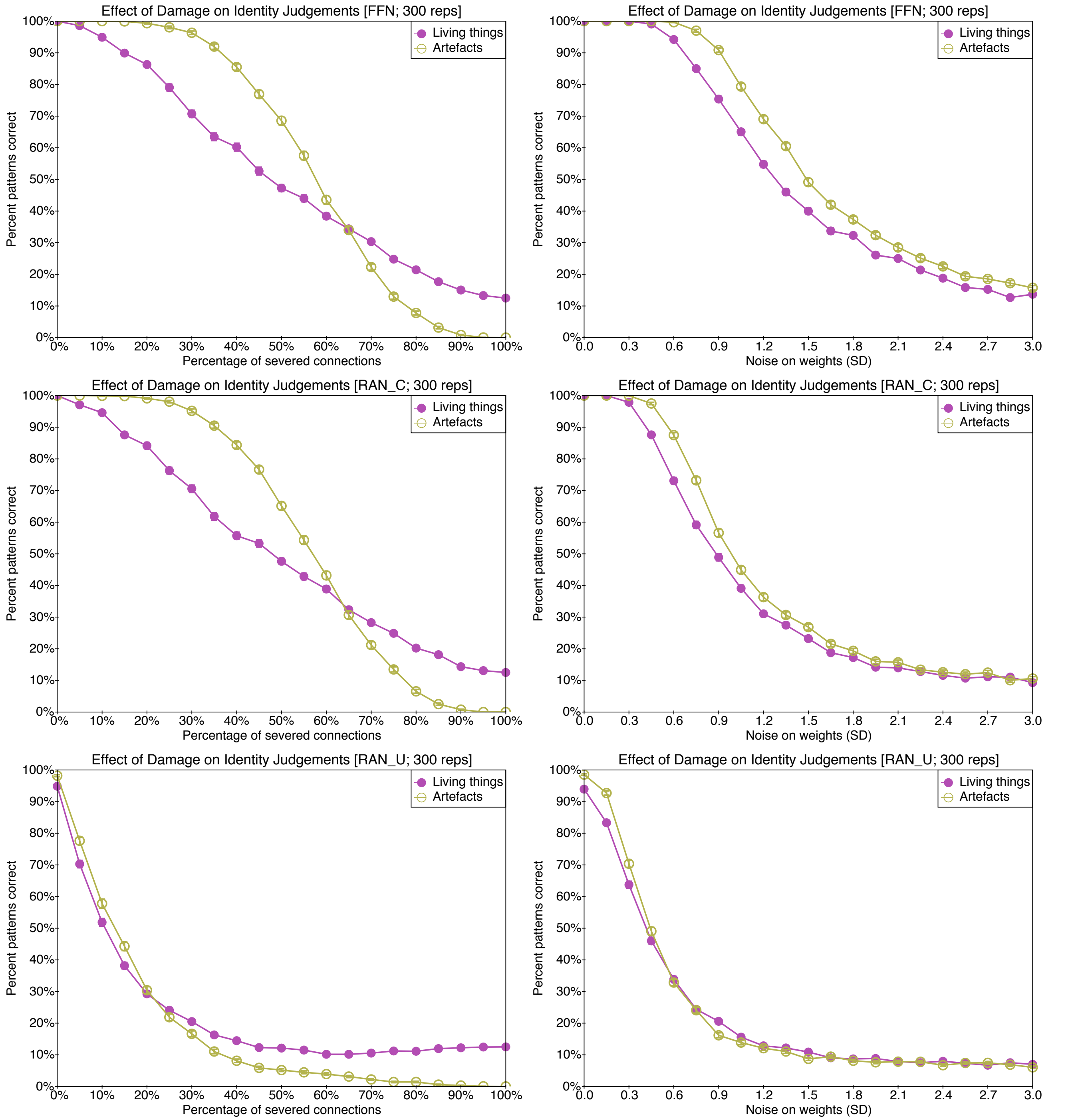

Figure 8. Accuracy of identity judgements for animals and artefacts in the feedforward network (FFN; upper), clamped recurrent attractor network (RAN_C; centre) and unclamped recurrent attractor network (RAN_U; lower) implementations of the conceptual structure model of Tyler et al. (2000). Left: Effect of lesioning connections. Right: Effect of adding noise to connection weights.

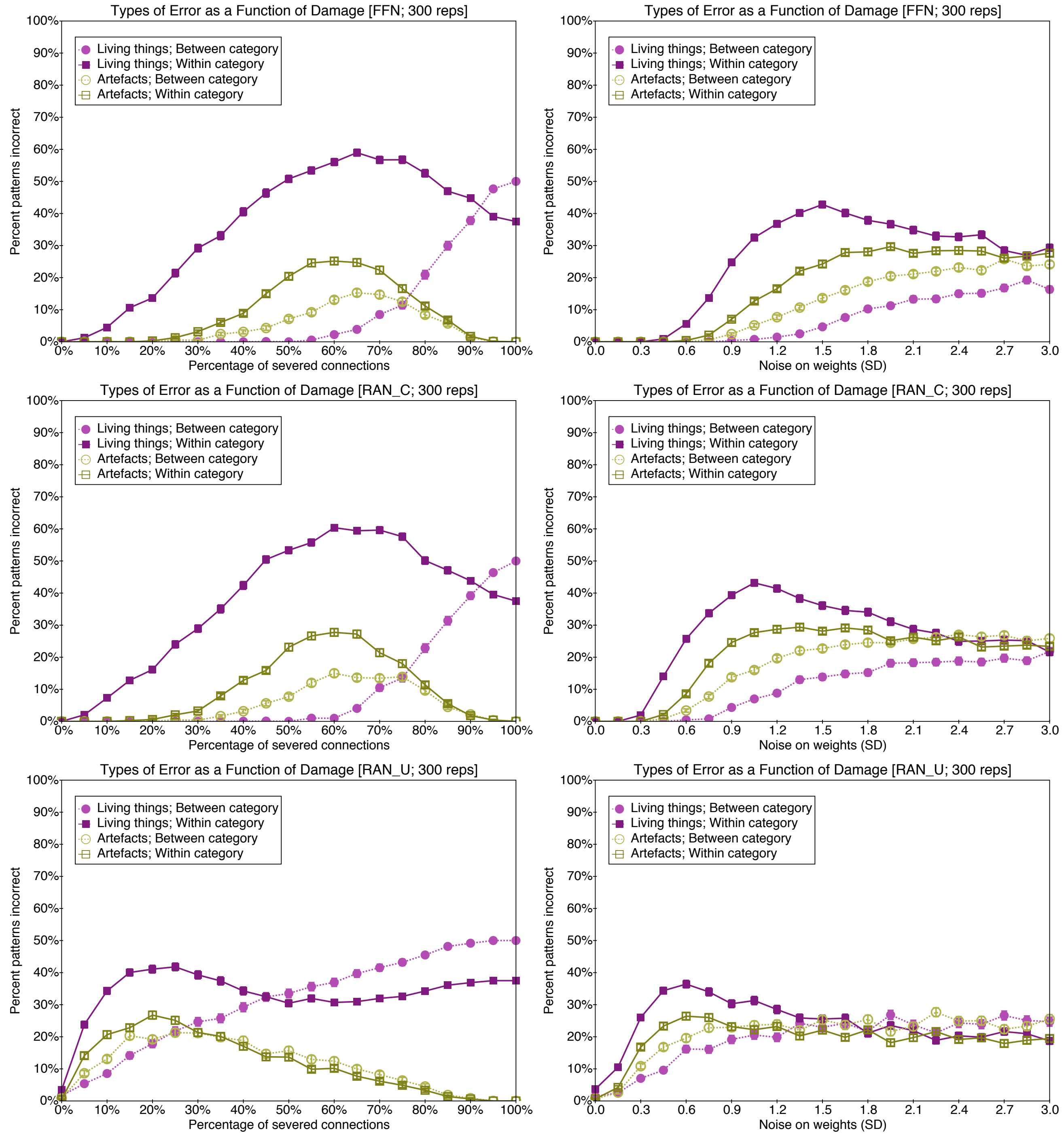

Figure 9. Analysis of error types by domain in the feedforward network (FFN; upper), clamped recurrent attractor network (RAN_C; centre) and unclamped recurrent attractor network (RAN_U; lower) implementations of the conceptual structure model of Tyler et al. (2000). Left: Effect of lesioning connections. Right: Effect of adding noise to connection weights.

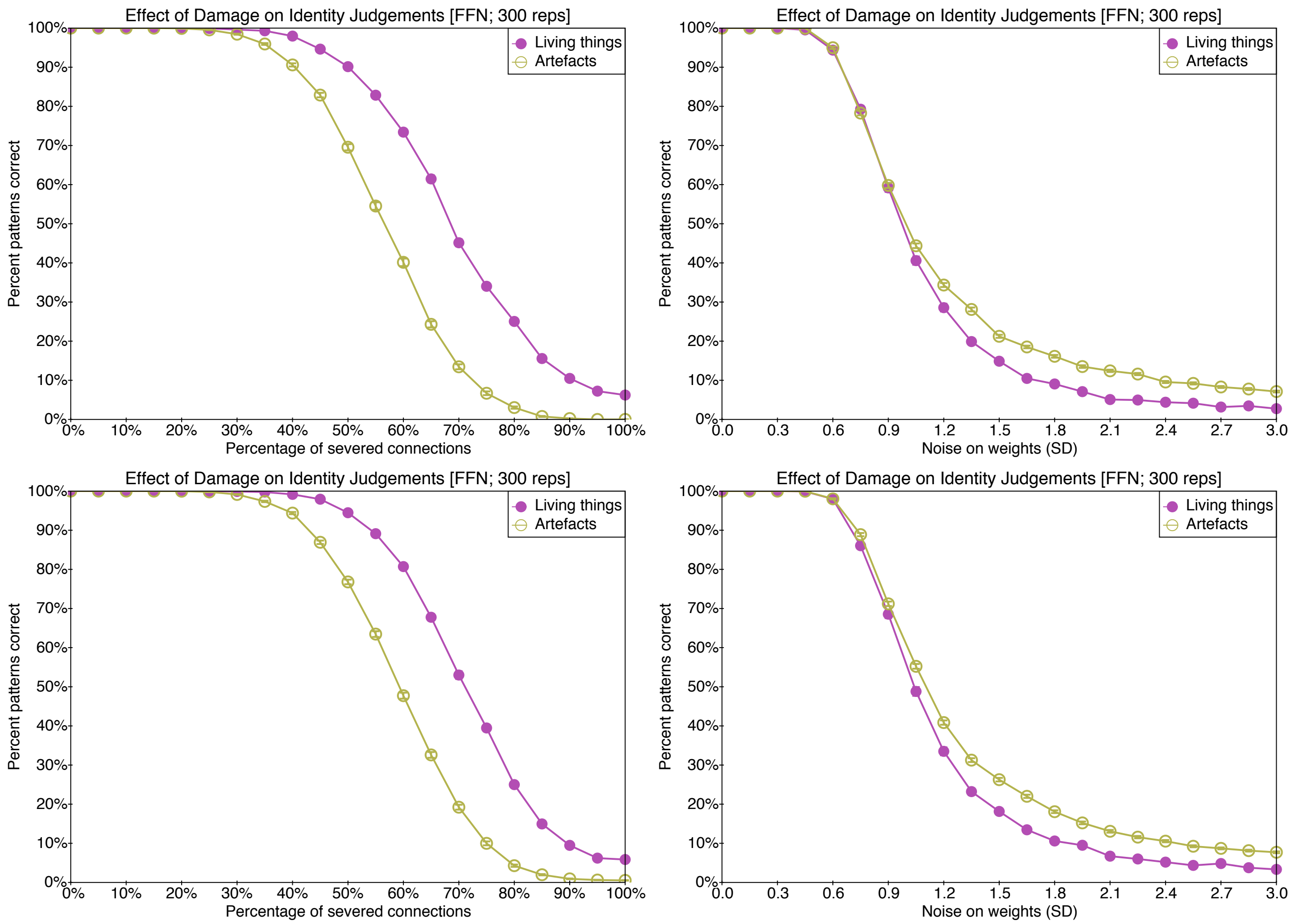

Figure 10. Accuracy of identity judgements (as assessed by the criterion of Tyler et al., 2000) for living things and artefacts in the feedforward auto-associative network when trained with pattern set P1 (upper) and when trained with pattern set P2 (lower). Left: Effect of lesioning connections. Right: Effect of adding noise to connection weights.

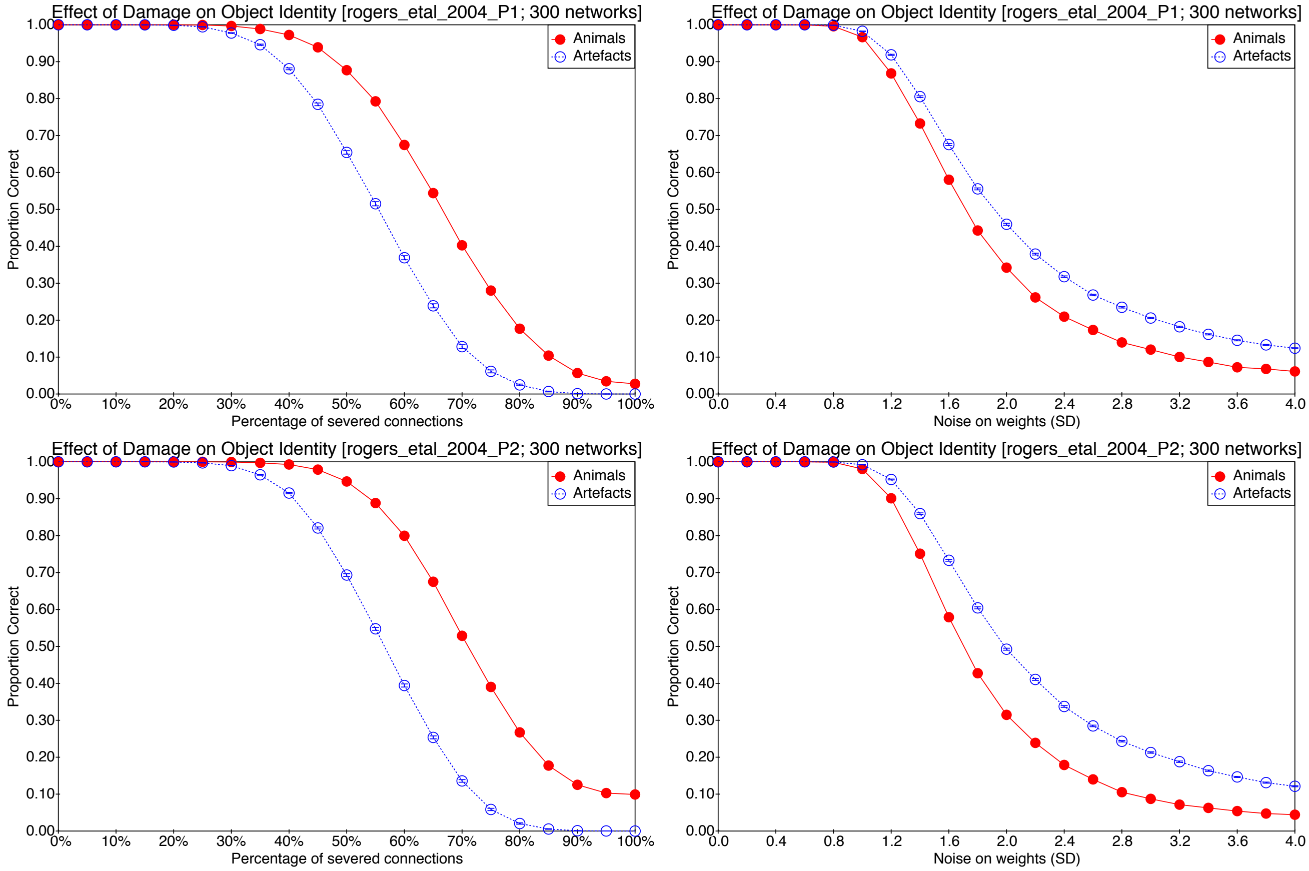

Figure 11. Accuracy of naming judgements (as assessed by the criterion of Lambon Ralph et al., 2007) for animals and artefacts in the feedforward autoassociative network when trained with pattern set P1 (upper) and when trained with pattern set P2 (lower). Left: Effect of lesioning connections. Right: Effect of adding noise to connection weights.

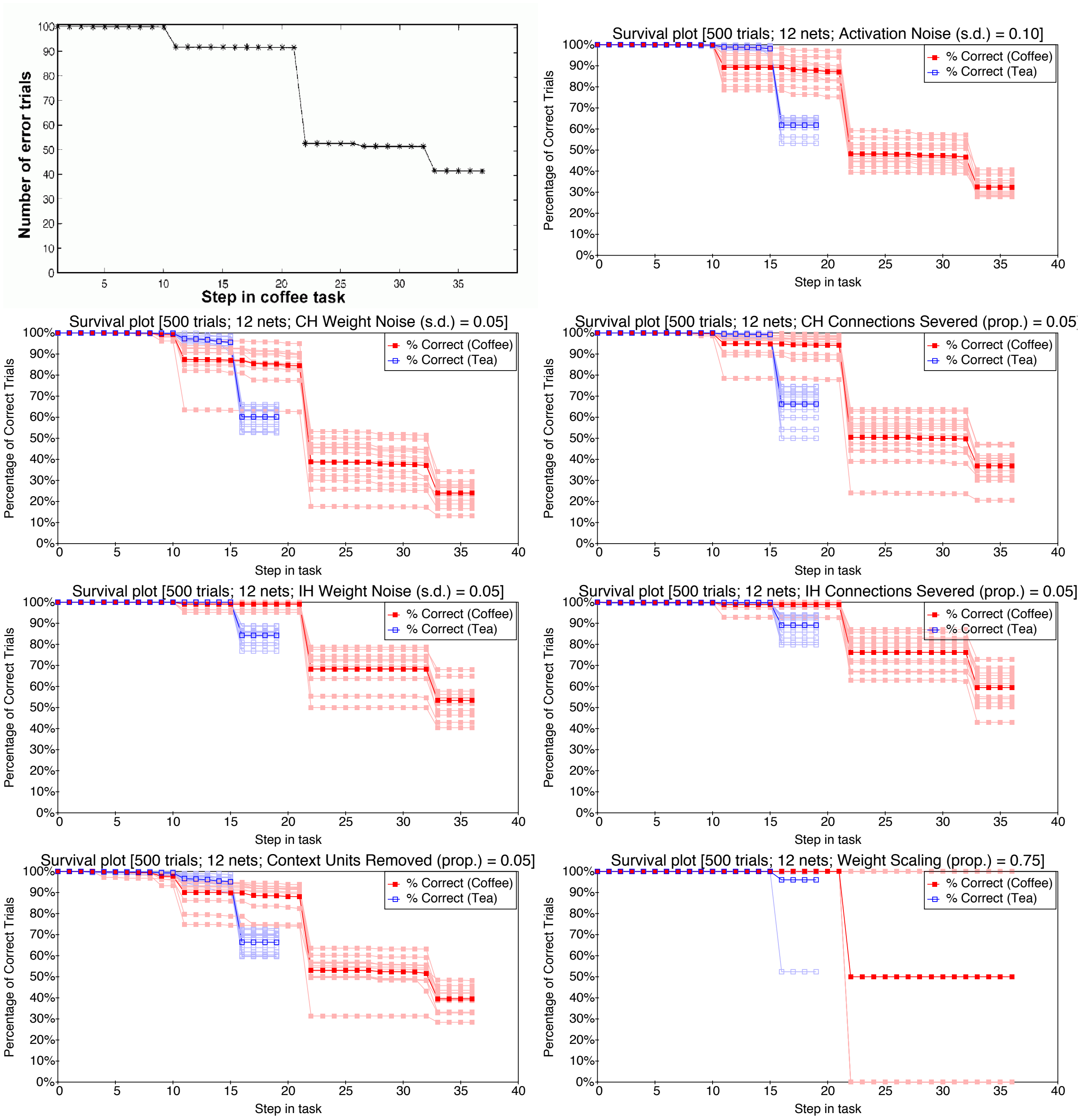

Figure 12. Survival plots for the original model, our replication, and six alternative approaches to modelling damage. The plots show the proportion of trials that are correct at each step of the task when the damaged model is attempting to prepare coffee (filled square markers) and tea (unfilled square markers). Upper row left: The original results for coffee preparation, as reported by Botvinick and Plaut (2004), when normally distributed noise (s.d. 0.10) was added to hidden unit activations. Upper row right: The direct (and successful) replication of Botvinick and Plaut (2004), extended to include the tea task. Second row left: The effect of normally distributed noise on context to hidden weights (s.d. = 0.05) on survival in both tasks. Second row right: The effect of lesioning connections between context and hidden units (at 5%) on survival in both tasks. Third row left: The effect of normally distributed noise on input to hidden weights (s.d. = 0.05) on survival in both tasks. Third row right: The effect of lesioning connections between input and hidden units (at 5%) on survival in both tasks. Lower row left: The effect of removing a proportion (5%) of context units on survival in both tasks. Lower row right: The effect of scaling weights (by 0.75) on survival in both tasks. All cases represent the results of 12 separately trained networks, differing only in the initial random values of weights.

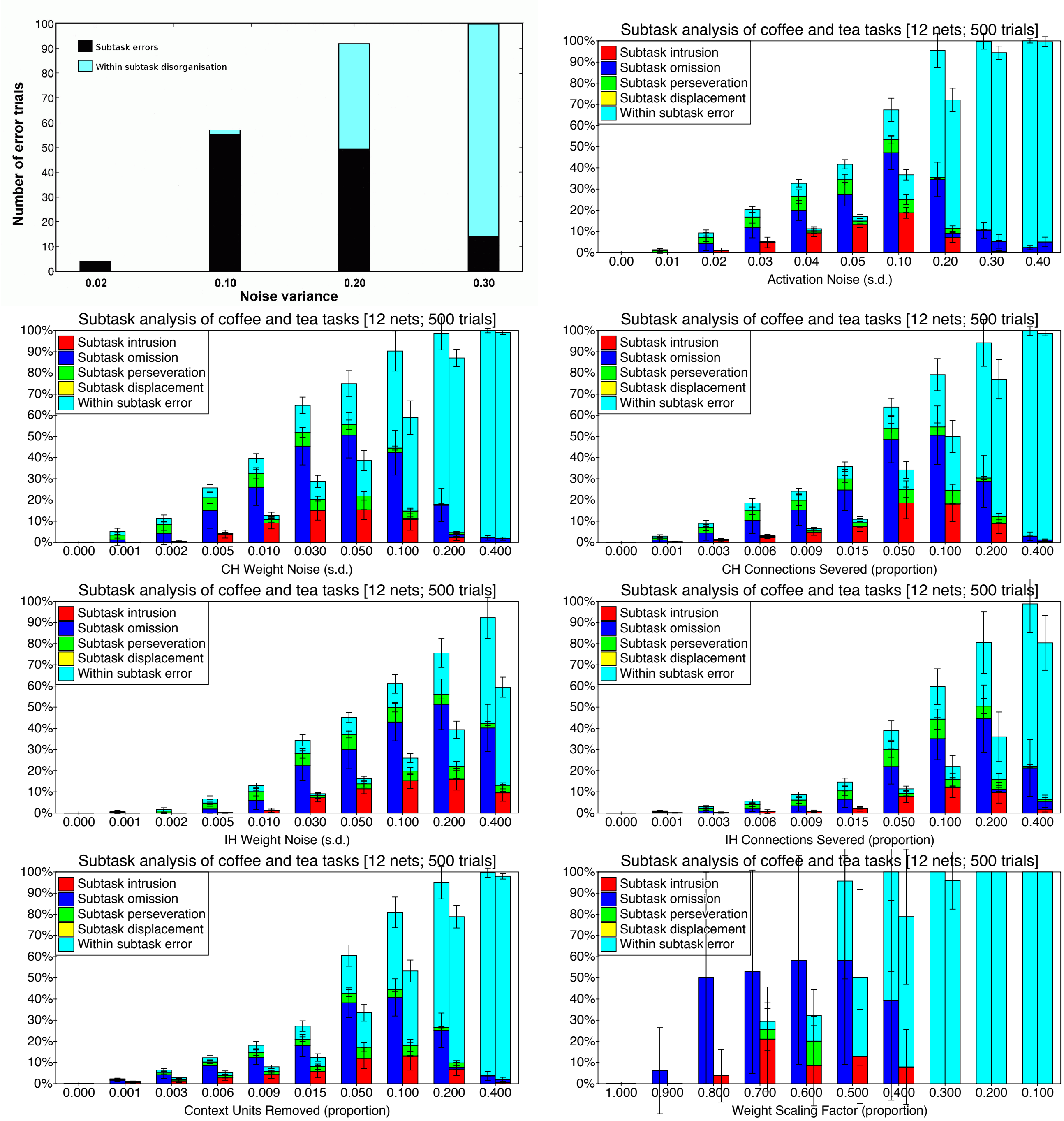

Figure 13. Error breakdown for the original model, our replication, and six alternative approaches to modelling damage. The plots show the proportion of trials containing a "subtask out-of-place" error versus a within-subtask error. Upper left: The original results for coffee preparation, as reported by Botvinick and Plaut (2004), when normally distributed noise was added to hidden unit activations. Upper right: The direct (and successful) replication of Botvinick and Plaut (2004), extended to include the tea preparation task. Second row left: The effect of normally distributed context-to-hidden weight noise on subtask errors in both tasks. Second row right: The effect of lesioning context-to-hidden connections on subtask errors in both tasks. Third row left: The effect of normally distributed noise added to input-to-hidden weights on subtask errors in both tasks. Third row right: The effect of lesioning input-to-hidden connections on subtask errors in both tasks. Lower row left: The effect of context unit removal on subtask errors in both tasks. Lower row right: The effect of weight scaling on subtask errors in both tasks. Where both tasks are shown on a graph, the left bars in each set relate to coffee preparation and the right bars in each set relate to tea preparation. All cases represent the results of 12 separately trained networks, differing only in the initial random values of weights, width error bars showing one standard deviation from the mean.

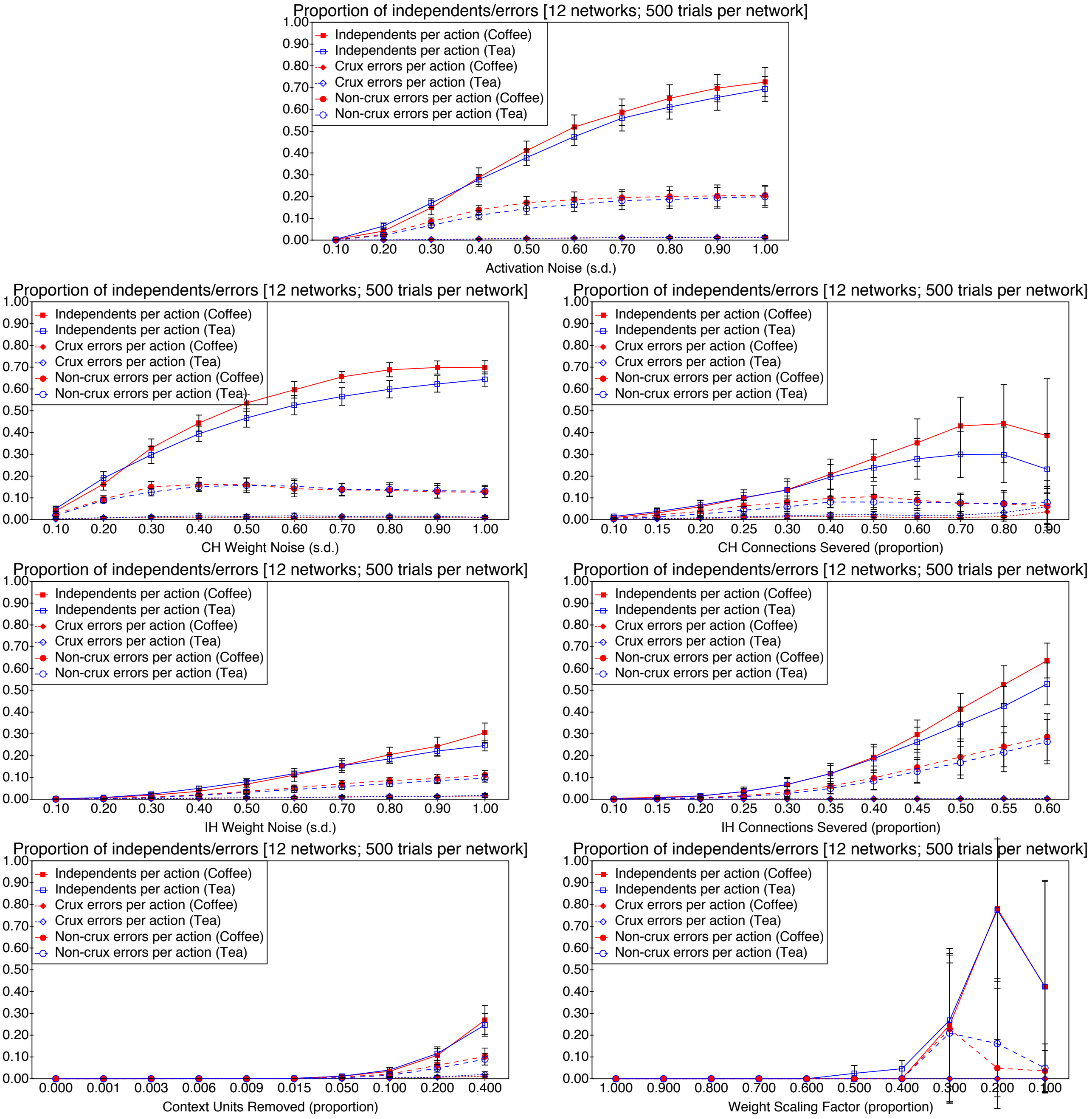

Figure 14. Error analysis for our replication of the original model, and six alternative approaches to modelling damage. The plots show the proportion of actions that are independent (i.e., not within the scope of subtask) and the proportion of actions that are crux and non-crux errors. Upper panel: The direct (and successful) replication of Botvinick and Plaut (2004), extended to include the tea preparation task. (No graph is shown from the original paper as Botvinick and Plaut (2004) did not report results for these simulations in graphical form.) Second row left: The effect of normally distributed weight noise on context-to-hidden units on subtask errors in both tasks. Second row right: The effect of lesioning context-to-hidden connections on subtask errors in both tasks. Third row left: The effect of normally distributed weight noise on input-to-hidden units on subtask errors in both tasks. Third row right: The effect of lesioning input-to-hidden connections on subtask errors in both tasks. Lower row left: The effect of removal of context units on subtask errors in both tasks. Lower row right: The effect of weight scaling on subtask errors in both tasks. All cases represent the results of 12 separately trained networks, differing only in the initial random values of weights, width error bars showing one standard deviation from the mean.

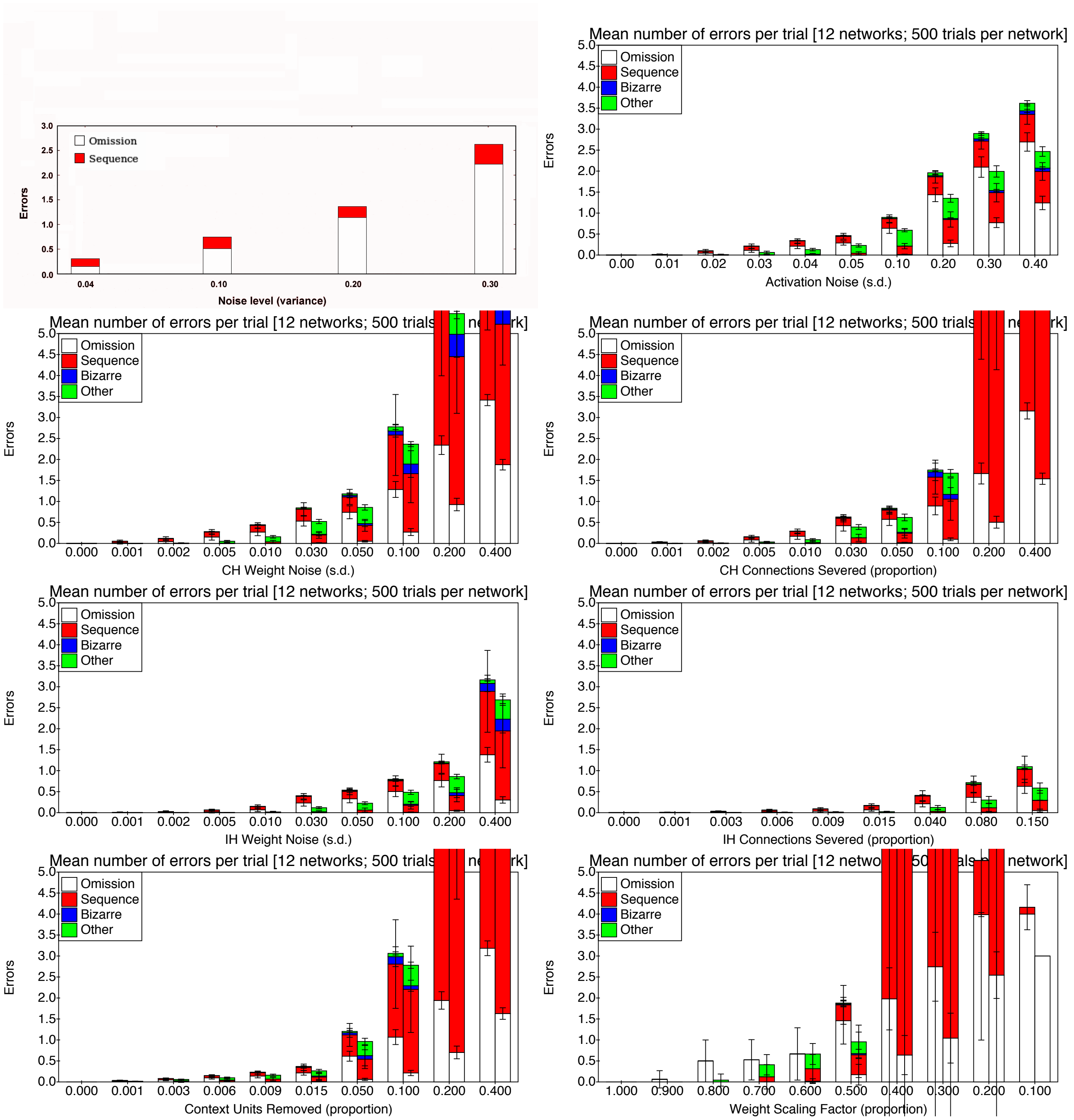

Figure 15. Number of errors per trial categorised by type for the original model, our replication, and six alternative approaches to modelling damage. Upper row left: The original results for coffee preparation, as reported by Botvinick and Plaut (2004), when normally distributed noise was added to hidden unit activations. Upper row right: The direct (and successful) replication of Botvinick and Plaut (2004), extended to include the tea preparation task. Second row left: The effect of normally distributed content-to-hidden weight noise on subtask errors in both tasks. Second row right: The effect of lesioning connections between context and hidden units on errors per trial in both tasks. Third row left: The effect of normally distributed input-to-hidden weight noise on subtask errors in both tasks. Third row right: The effect of lesioning input-to-hidden connections on errors per trial in both tasks. Lower row left: The effect of context unit removal on subtask errors in both tasks. Lower row right: The effect of weight scaling on errors per trial in both tasks. Where both tasks are shown on a graph, the left bars in each set relate to coffee preparation and the right bars in each set relate to tea preparation. All cases represent the results of 12 separately trained networks, differing only in the initial random values of weights, width error bars showing one standard deviation from the mean.
